# Poly (I:C)-induced inflammation requires the activation of toll-like receptor 3/Ca^2+^/CaMKII/pannexin 1-dependent signaling

**DOI:** 10.7150/thno.100687

**Published:** 2025-01-20

**Authors:** Magdiel Salgado, Vania Sepúlveda-Arriagada, Macarena Konar-Nié, María A. García-Robles, Juan C. Sáez

**Affiliations:** 1 Instituto de Neurociencias, Centro Interdisciplinario de Neurociencias de Valparaíso, Universidad de Valparaíso, Valparaíso, Chile.; 2 Facultad de Medicina y Ciencia, Universidad San Sebastián, sede Concepción, Chile.; 3 Departamento de Biología Celular, Facultad de Ciencias Biológicas, Universidad de Concepción, Concepción, Chile.; 4 Departamento de Ciencias Biológicas, Facultad de Ciencias de la Vida, Universidad Andrés Bello, Concepción, Chile.

**Keywords:** Panx1, hemichannel, pannexon, Poly (I:C), viral infections

## Abstract

Pannexin1 (Panx1) is a glycoprotein, ubiquitously expressed throughout vertebrate tissues. In the cell membrane, it forms non-selective hemichannels (Panx1 HCs) that allow the release of ATP. This extracellular ATP triggers purinergic signaling relevant to the immune responses to pathogens, including viruses. While the activity of Panx1 HCs is known to be elevated by some viruses, the underlying molecular mechanism remains elusive.

**Methods**: In this study, we used Poly(I:C), a double-stranded RNA analog that constitutes a hallmark of viral infections. Peritoneal macrophages were obtained from wild-type and Panx1 knock-out mice. The mRNA levels of proinflammatory cytokines were quantified by RT-qPCR. We also evaluated hemichannel activity through dye uptake assays, whereas Ca^2+^ signals were studied using Fura-2 and GcamP6. Panx1-P2X_7_R interaction was studied by proximity ligation assays.

**Results:** Panx1 expression and activity were crucial for the proinflammatory response induced by Poly(I:C) in RAW264.7 cells and peritoneal macrophages. In HeLa cells transfected with mPanx1 (HeLa-mPanx1) and RAW264.7 cells, Poly (I:C) increased Panx1 HC activity in a concentration-dependent manner, which was inhibited by ^10^Panx1, a peptide that selectively blocks Panx1 HCs. Furthermore, the Poly(I:C)-induced rise in Panx1 HC activity correlated with a rapid increase in intracellular Ca^2+^ signal, dependent on TLR3 and P2X_7_R activity. Interestingly, lasting exposure to Poly (I:C) promoted the interaction and internalization of the Panx1-P2X_7_R complex, which depended on CaMKII, Panx1 HC, and P2X_7_R activities. The Poly (I:C)-induced increase in Panx1 HC activity was entirely prevented by Ca^2+^ chelation with BAPTA-AM, CaMKII blockage with KN-62, or PKA activation with db-cAMP. These findings were consistent with data from Panx1 mutants that either avoid or mimic phosphorylation at kinase target sites. Supporting this finding, we demonstrated that CaMKII activity is essential for the inflammatory response triggered by Poly (I:C) in macrophages.

**Conclusion**: A TLR3/Ca^2+^/CaMKII/Panx1 HC pathway is crucial in orchestrating the cellular response to viral patterns and presents a potential novel target for preventing infections and alleviating the harmful effects associated with RNA-based viral infections.

## Introduction

Pannexins (Panxs) constitute a family of integral membrane proteins comprising three members (Panx1, 2, and 3), of which Panx1 is the most ubiquitous 1. Panx1 assembles into hemichannels (Panx1 HCs), through the aggregation of seven protein subunits, forming a structure also known as pannexon 2. Panx1 HCs are characterized by low selectivity and a unitary conductance of ~75 ps 2. One of the most studied functions of these hemichannels is the release of ATP as an autocrine or paracrine signal, given that extracellular ATP affects the same cell or neighboring cells through interactions with ionotropic (P2XRs) or metabotropic (P2YRs) purinergic receptors 3. While low extracellular ATP concentration predominantly activates a P2YRs-dependent G protein-coupled transduction pathway, followed by IP_3_ formation that triggers Ca^2+^ release from intracellular reservoirs, high ATP concentrations activate P2XRs causing an influx of calcium ions (Ca^2+^). In this context, ATP-dependent signaling has been implicated in a variety of physiological and pathophysiological cell responses 4, 5.

In recent years, the role of purinergic signaling in various immune responses has been of particular interest. Particularly, the interaction of Panx1 HC with P2X_7_ receptor (P2X_7_R) has been shown to promote an immune response mediated by the release of IL-1β in response to ATP, which occurs after caspase-1 activation 6. It has also been proposed that Panx1 HCs participate in the activation of NLRP1 or NLRP3 inflammasome in diverse cell types including macrophages, neurons, and astrocytes, a process that also depends on the P2X_7_R 6-8, leading to cell death by apoptosis 9-11. Other functions of Panx1 HCs include favoring the activation of CD4^+^ T lymphocytes through a P2X_1/4_R- and MAPK-dependent mechanism 12, 13, participating in the activation of the endothelium in the event of infection to enhance diapedesis of leukocytes 14, accelerating dendritic cell migration 15, and regulating trafficking of pathogenic patterns from endosomes to the cytosol in response to ATP 16.

Regarding viral infections, a growing amount of evidence shows that purinergic signaling plays a critical role in favoring infection by and/or replication of different types of viruses in human cells. For example, extracellular ATP plays a key role in HIV infection in CD4^+^ cells 17, and this response depends on Panx1 HCs 18. In the case of bronchoalveolar epithelium infection with the respiratory syncytial virus (RSV), UTP is released and acts autocrinally on P2Y receptors, reducing alveolar fluid clearance and causing pneumonia 19. Using *in vitro* models, it has been shown that activation of toll-like receptors 3 or 4 (TLR3 or TLR4) mediates increases in intracellular Ca^2+^ concentration ([Ca^2+^]_i_) in an IP_3_ receptor-dependent manner, which is necessary to increase the expression and release of cytokines 20. Since the increase in [Ca^2+^]_i_ is a classical stimulus to activate Panx1 HCs 21, 22, we wonder if virus-mediated activation of TLR3 can modulate Panx1 HC activity.

Here, we used Poly (I:C), a mismatched double-stranded RNA that mimics the genome of many RNA-based viruses, and whose main endogenous target is TLR3 to activate an innate immune response. We demonstrated that this compound increases the activity of Panx1 HCs, through a mechanism that depends on Ca^2+^ mobilization from reticular sources and post-translational modifications of the C-terminal tail of Panx1. Moreover, Panx1 HC activity was necessary for evocating a purinergic-dependent pro-inflammatory response, providing a therapeutic potential to regulate the harmful effects of massive immune responses.

## Material and methods

**Reagents and antibodies.** Poly (I:C) of high molecular weight (1.5-8 kb) was obtained from InvivoGen (San Diego, USA). Db-cAMP, carbenoxolone, TLR3i (a TLR3 inhibitor), KN-62, Duolink® In Situ Red Starter Kit Mouse/Rabbit, xestospongin C, poly-lysine, KN-62, suramin, db-cAMP, PKAi, and A23187 were obtained from Sigma-Aldrich (St. Louis, Missouri, USA). Alexa fluor 488-conjugated WGA, BAPTA-AM, and Fura-2 AM were obtained from ThermoFisher Scientific (Waltham, Massachusetts, USA). D4, a selective connexin hemichannel blocker, was obtained from EDELRIS s.a.s. (Lyon, France). ^10^Panx1, A740003, and DAPI were purchased from Tocris Bioscience (Bristol, United Kingdom). Anti-Iba1, anti-P2X_7_R, and anti-TLR3 antibodies were from Abcam (Cambridge, United Kingdom). Anti-α-tubulin was from Santa Cruz Biotechnology (Dallas, Texas, U.S.A.). Rabbit anti-N-terminal Panx1 (Abcam) and homemade rabbit anti-C-terminal Panx1 were also used. Anti-rabbit or anti-mouse IgG (Jackson ImmunoResearch, City, USA) were used as secondary antibodies.

**Cell cultures.** Parental HeLa cells (HeLa-P) were obtained from the American Type Culture Collection (ATCC, Rockville, MD) and cultured as previously described 23. The identity of HeLa cells and their derivatives was confirmed by STR profiling, and phase-contrast cell images as described previously 24. HeLa-P cells or 24-h transfected with either the mPanx1 fused to GFP (HeLa-mPanx1), hPanx1 fused to mCherry (HeLa-hPanx1), or rPanx1 mutants were grown in DMEM low glucose medium. RAW264.7 cells were also obtained from the ATCC and cultured in a DMEM high-glucose medium. All cell types were grown in the corresponding medium supplemented with 10% fetal bovine serum and 50 U/mL penicillin-streptomycin, maintained at pH 7.4 at 37 °C in an incubator with a 5% CO_2_/95% air atmosphere. Cells were passaged upon reaching 70-80% confluence. When it was required to maintain stable transfection, cells were cultured with 1 mg/mL G418 (Invitrogen, Waltham, MA, USA) from 24 h post-transfection.

**Generation of pannexin 1 knock out RAW cells.** Panx1 knock-out (Panx1^-/-^) RAW cells were generated using the CRISPR/Cas9 technique. In brief, RAW264.7 cells were transfected with an all-in-one vector targeting Panx1 exon 2 (PANX1 CRISPR gRNA1_pSpCas9 BB 2A-Puro (PX459) v2.0, SC1678, GenScript Biotech, China). After 24 h, clone selection was performed using 5 µg/mL puromycin and replacing the medium every other day until all cells died in the control plates (non-transfected). Complete Panx1 knock-out in RAW cells was demonstrated by Western blot analysis after 2 weeks of culture without antibiotics.

**Dye uptake**. The functional state of Panx1 HCs was evaluated in dye-uptake experiments with DAPI, which were performed as previously described 23. Cells were washed twice in PBS and then exposed to Krebs solution (containing: 154 mM NaCl, 5.4 mM KCl, 2.3 mM CaCl_2_, 5 mM HEPES, and pH 7.4) with 5 µM DAPI, and time-lapse measurements of fluorescent intensity were recorded for each experimental condition over a 5-min period. Dye uptake rates were calculated from the slopes of the curve for each condition.

**Intracellular Ca^2+^ signal.** HeLa-mPanx1-YFP and RAW cells were used to determine changes in cytoplasmic Ca^2+^ in response to Poly(I:C) as previously described 25. In brief, cells were incubated in Krebs-Ringer buffer containing Fura-2 AM (5 μM) for 30 min at 37 °C followed by a de-esterification period of 10 min at 37 °C. Then, the Ca^2+^ signal was recorded every 3 s using a Nikon Eclipse Ti microscope (Tokyo, Japan) at two excitation wavelengths (340 and 380 nm) to calculate the ratio of the recorded fluorescence emissions called Ca^2+^ signal (340/380 ratio). In addition, sub-membranous Ca^2+^ influx was evaluated in cells transfected with genetically engineered Ca^2+^ sensor Lck-GcamP3 (plasmid #26974) (Addgene, Watertown, MA, USA), which allowed time-lapse recordings of Ca^2+^ uptake (ex/em of 480/510 nm). Data was collected every 3 s in an inverted fluorescence microscope and the values were plotted as ΔF/F_o_ over time**.**

**Proximity Ligation Assays (PLA)**. To assess endogenous interactions between Panx1 and P2X_7_R in RAW cells, proximity ligation assays were performed following the manufacturer's instructions (Sigma-Aldrich). To this end, 1x10^5^ RAW cells were seeded onto 12 mm glass coverslips coated with poly-lysine. After applying each stimulus, cells were fixed with 4% PFA, stained with Alexa fluor 488-conjugated WGA, permeabilized, and then incubated for 16 h with anti-Panx1 and anti-P2X_7_R antibodies (from mouse and rabbit, respectively). Subsequently, cells were incubated for 1 h at 37 °C with a mixture consisting of 8 μL of each PLA probe and were then ligated using 1 μL of ligase and 1X ligation buffer for 30 min at 37 °C. Finally, the amplification was carried out using 0.5 μL of polymerase for 100 min at 37 °C. Images were captured on a Nikon confocal microscope with a 100X objective. We used ImageJ/Fiji software for image processing, as previously reported 26. In our analysis, a PLA event was defined as a red dot of approximately 0.5-1 µm in diameter.

**Co-immunoprecipitation**. Panx1-P2X_7_R interaction was also evaluated by co-immunoprecipitation. For this, 3x10^6^ RAW cells were seeded in 6-well plates and incubated with Poly (I:C), ATP, or Poly (I:C) + ^10^Panx1 for 30 min. After that, cells were lysed with coIP lysis/wash buffer (in mM, Tris 25, NaCl 150, EDTA 1; in % NP-40 1 and glycerol 5) and proteins were quantified through the Bradford method. 250 µg of proteins from each condition were incubated overnight at 4 °C with 1 µg mouse anti-P2X_7_R antibody and next incubated for 2 h at 4 °C with 30 µL protein A/G plus agarose (Santa Cruz Biotechnology, cat # sc-2003). Precipitated proteins were then denatured and loaded in SDS-PAGE for western blot evaluation of Panx1 interaction.

**Western blot analysis**. For protein analysis, cells were homogenized in a M-PER mammalian protein extraction buffer supplemented with a complete protease and phosphatase inhibitor cocktail (ThermoFisher Scientific), and then sonicated three times on ice at 300 W (Sonics & Material INC, VCF1, Connecticut, USA) for 10 s. After centrifugation at 8,000× g for 10 min, the suspended proteins were quantified and resolved by SDS-PAGE (50 μg/lane) in a 10% (w/v) polyacrylamide gel, transferred to nitrocellulose membranes and probed with a rabbit anti-TLR3 (1:1,000), a rabbit anti-Panx1 (1:2,000) or mouse anti-α tubulin (1:5,000) antibodies. After extensive washes, the membranes were incubated for 2 h at 4 °C with peroxidase-labeled anti-rabbit or anti-mouse IgG (1:5,000). The reaction was developed using an enhanced chemiluminescence (ECL) Western blot analysis system (Amersham Biosciences, Amersham, United Kingdom). Negative controls consisted of incubating the membrane in the absence of primary antibodies.

**Quantification of mRNA levels.** RT-qPCR analysis was used to quantify TNF-α, IL-1β, Panx1, and GAPDH (the housekeeping gene) mRNA levels. Total RNA was isolated using TRIzol (Invitrogen) and treated with DNase I (ThermoFisher Scientific) to eliminate any potential genomic DNA contamination. A total of 0.5 μg of RNA from each sample was reverse transcribed into cDNA according to the manufacturer's protocol of Verso cDNA Synthesis Kit (ThermoFisher Scientific). RT-qPCR reactions were prepared with the KAPA SYBR® FAST qPCR master mix (Hoffman-La Roche, Basilea, Switzerland) in a final volume of 20 μL, containing 1x SYBR Green Master Mix, 1 μL cDNA sample (1:10) and 200 nM of the following primer sets: TNF-α: Fwd: 5'- CGC TCT TCT GTC TAC TGA AC-3', Rev: 5'-TGT CCC TTG AAG AGA ACC TG-3'; IL-1β: Fwd: 5'- TGG GAT GAT GAT GAT AAC CT -3', Rev: 5'-CCC ATA CTT TAG GAA GAC AGG GAT TT-3'; Panx1: Fwd: 5'-CTC GGA CTT CTT GCT GAA GG-3', Rev: 5'-CTT AAT CAT GCC CAG GTT TGT C-3'; TLR3: Fwd: 5'-AAC TTA GCA CGG CTC TGG AAA CAC-3', Rev: AAA GCT GGC CCG AAA ACC TTC T-3'; GAPDH: Fwd: 5'-CTG AGT ATG TCG TGG AGT CTA-3', Rev: 5'-GAT GCA GGG ATG ATG TTC TG-3'. All reactions were performed with an initial denaturation of 5 min at 95 °C, followed by 40 cycles of 30 s at 95 °C, and annealing for 30 s at 60 °C in a PikoReal™ Real-Time PCR System (ThermoFisher Scientific™). C_t_ values of each cDNA resulting from three different experiments were normalized using the 2^-ΔΔCt^ method, with GAPDH serving as the reference gene.

**Immunocytochemistry.** Cultured cells were grown on poly-L-lysine coated (Sigma-Aldrich) glass cover slides in 24-well plates, fixed with 4% PFA in PBS for 30 min, washed with Tris-HCl buffer (pH 7.8) and incubated in the same buffer containing 1% BSA and 0.2% Triton X-100 for 10 min at RT. Samples were then incubated with primary antibodies, followed by incubation with Cy^2^- and/or Cy^3^-labeled secondary antibodies (Jackson ImmunoResearch Laboratories), counterstained with the DNA stain DAPI (1:1,000; Invitrogen), and analyzed using confocal laser microscopy (Carl Zeiss, LSM700).

### Quantification of extracellular ATP levels

RAW264.7 cells were seeded in 24-well plates, and the culture medium was replaced 24 h later (80% confluency). Two hours later, cells were treated for 30 min with 10 µg/mL Poly (I:C) or in combination with 200 µM ^10^Panx1. Alkaline solution (pH 8.5) was also assayed as a positive control of hemichannel activation in the cell membrane. ATP concentration in the extracellular solution was measured using a luciferin/luciferase bioluminescence assay kit (ThermoFisher Scientific). Baseline measurements were performed on separate cultures using standard Krebs's solution. The amount of ATP in each sample was calculated from standard curves and normalized for the protein concentration using the Bradford assay (Bio-Rad Laboratories, Hercules, CA USA).

**Peritoneal macrophages extraction.** All mice (C57BL/6, males, 2-month-old, weighing 20-25 g) were handled in accordance with instructions of the Animal Welfare Assurance. All animal work was approved by the appropriate Ethics and Animal Care and Use Committee at Universidad de Concepción, Chile (permit number 2010101 A). The mice had free access to a standard rodent diet (Lab Diet, 5P00 Prolab RMH 3000, Purina Mills, St. Louis, MO) and tap water. Peritoneal macrophage extraction was performed as described 27. Particularly, 30 µg Poly (I:C) or PBS (100 µL each) were intraperitoneally injected in wild-type (WT) or Panx1^-/-^ mice. After 12 h of treatment, mice were euthanized, and 5 mL of PM buffer (containing PBS + 30% FBS) was applied intraperitoneally with a 27G syringe through a ventral window. The abdomen of each animal was carefully massaged, and the peritoneal fluid was transferred to a tube completing 30 mL of PM buffer. The cells were centrifuged at 500 g for 10 min, resuspended in RPMI medium, and seeded onto a 24-well plate for 2 h before further processing.

**Statistical analysis**. Student's two-tailed, paired t-test was used to compare two different groups. When analyzing more than two groups, we used a two-tailed ANOVA. A p-value of ≤ 0.05 was considered statistically significant. All statistical analyses were performed using GraphPad Prism 8.5 software.

## Results

### Pannexin1 expression is necessary for Poly (I:C)-induced inflammation

**Figure 1:**
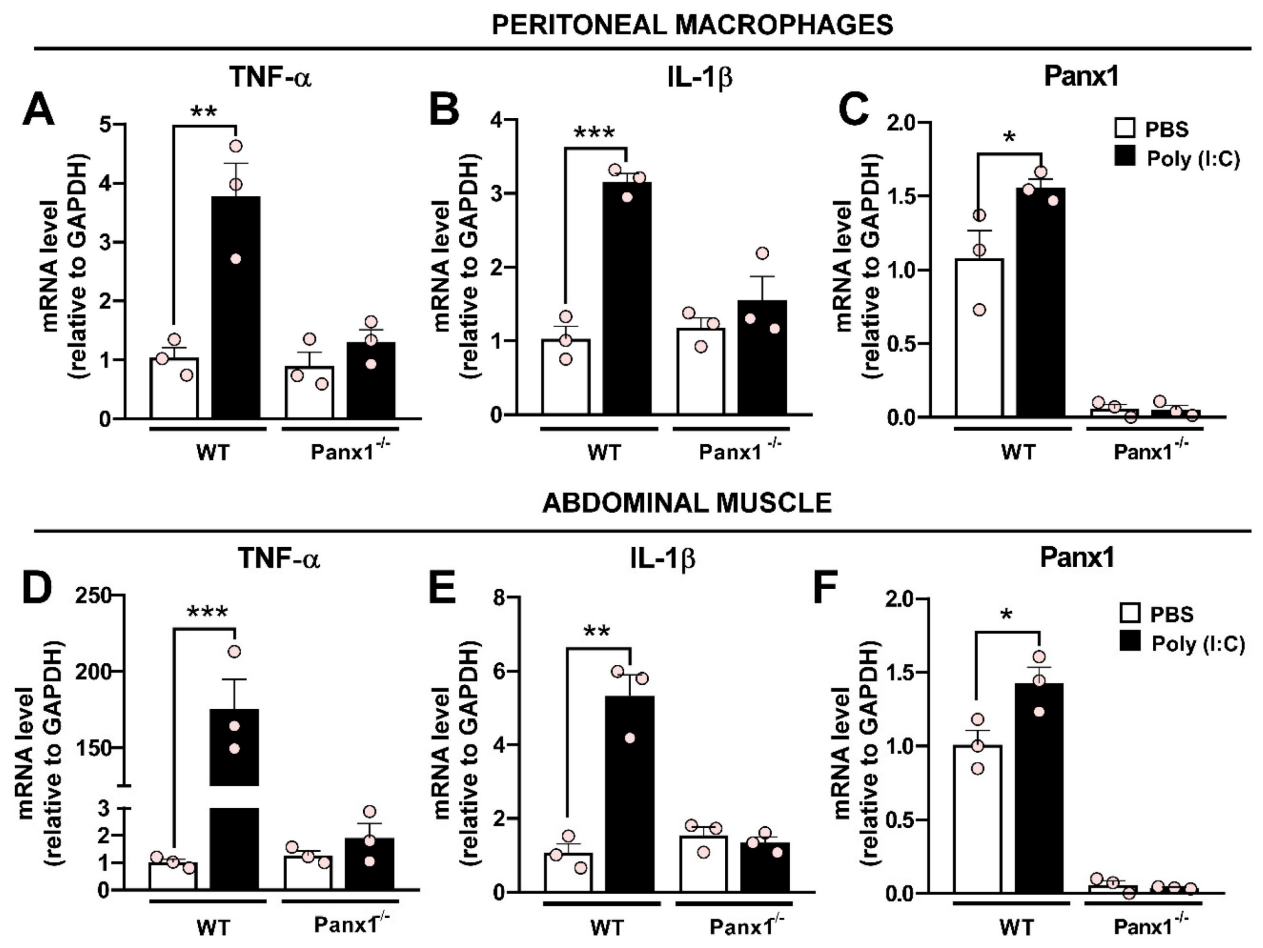
** The Poly (I:C)-induced proinflammatory response depends on pannexin1 expression**. **A-C**. mRNA levels of TNF-α (A), IL-1β (B), or Panx1 (C) in peritoneal macrophages from WT or Panx1^-/-^ mice after 12 h of i.p.-injected with PBS or 30 µg Poly (I:C) (n = 3). **D-F**. mRNA levels of TNF-α (A), IL-1β (B) or Panx1 (C) in abdominal muscle from mice described above (n = 3). Each point represents the mean ± SEM. one-way ANOVA followed by Tukey post hoc test. * p < 0.05; ** p < 0.01; *** p < 0.001.

The pro-inflammatory effect of Poly (I:C) has been widely established 28, 29. However, the contribution of Panx1 HCs in this response remains unknown. To investigate this, we carried out experiments in which wild-type (WT) or Panx1^-/-^ mice were intraperitoneally injected once with 30 µg Poly (I:C)-fluorescein or PBS, and sacrificed after 12 h, as previously described 30. Immediately, peritoneal macrophages (PMs) were extracted and cultured for 2 h before processing for immunofluorescence or RT-qPCR. Immunofluorescence analysis revealed that Poly (I:C) was uniformly distributed throughout enlarged WT PMs, which were positive for Iba1 and Panx1 (Figure S1). Furthermore, Poly (I:C) induced Panx1 internalization in WT PMs. By contrast, a very weak Poly (I:C)-associated signal was detected in Panx1^-/-^ PMs, suggesting that Panx1 is a critical factor in Poly (I:C) endocytosis. RT-qPCR analysis showed that WT PMs respond to Poly (I:C) challenges with upregulation of TNF-α (3.8-fold, p = 0.0018 vs. PBS) and IL-1β (3.2-fold, p = 0.0003 vs. PBS) mRNA levels. However, no significant changes in mRNA levels of these cytokines were found in Panx1^-/-^ PMs in response to Poly (I:C) (Figure 1A-B). Furthermore, Poly (I:C) injection evoked a significant increase of Panx1 mRNA in WT PMs (1.56 ± 0.05, p = 0.0378 vs. PBS) (Figure 1C). In the same way, in abdominal muscle samples from WT mice, we found a very high response to Poly (I:C) injection, evoking a 175-fold increase for TNF-α (174.7 ± 19.2, p = 0.0003) and 5-fold increase for IL-1β (5.32 ± 0.57, p = 0.0007) mRNAs, with respect to PBS injection (Figure 1D-E). However, in Panx1^-/-^ mice, mRNA levels of these cytokines were unaffected by Poly (I:C). As in PMs, mRNA levels of Panx1 significantly increased in abdominal muscle after 12 h of Poly (I:C) injection (Figure 1F). However, mRNA levels of TNF-α, IL-1β or Panx1 were not affected by Poly (I:C) in the brain cortex or lungs, neither from WT nor Panx1^-/-^ mice (Figure S2). Thus, Panx1 appears to be crucial to evoke a local proinflammatory response in mice.

### Poly (I:C) activates pannexin1 hemichannels involved in inflammation

**Figure 2:**
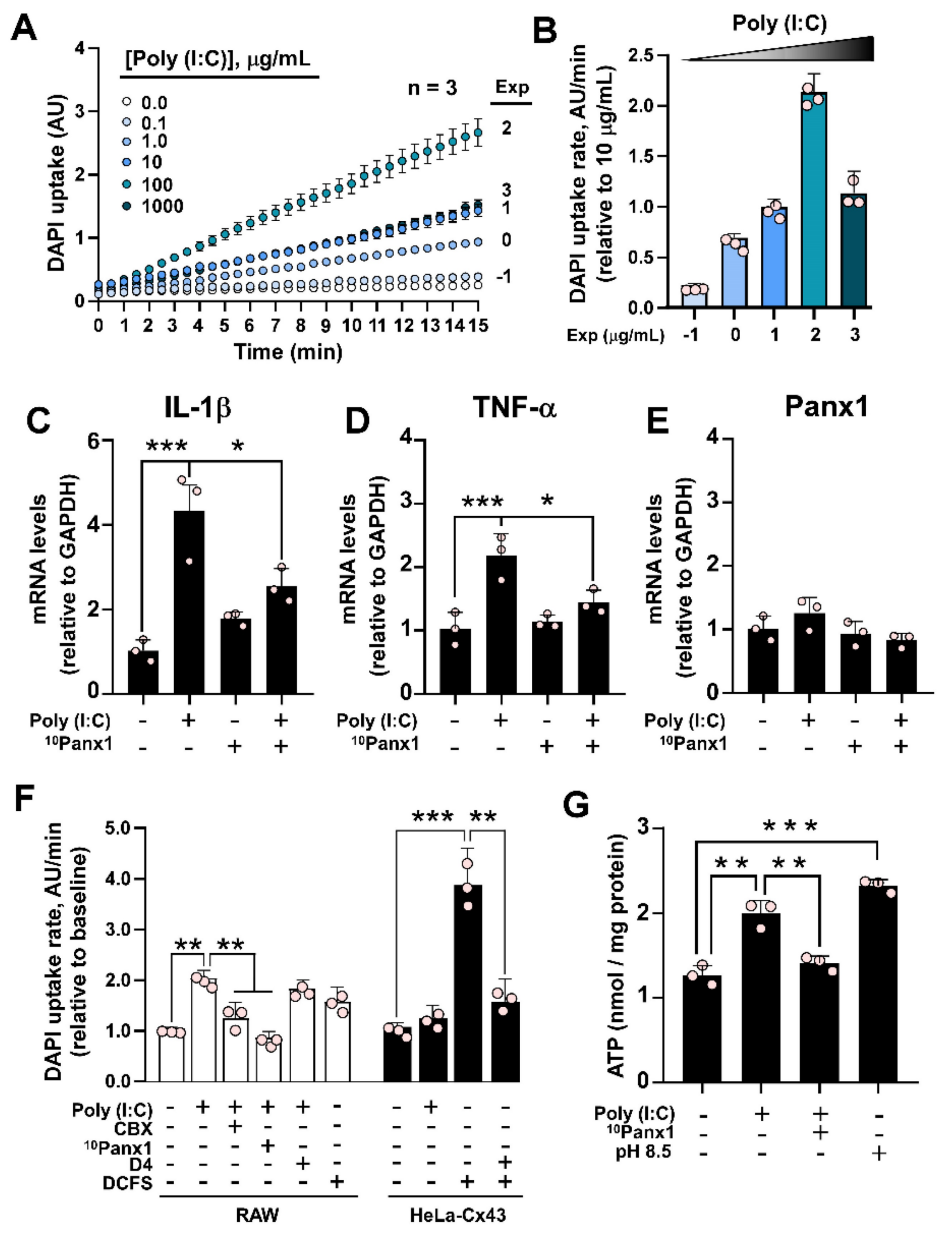
**Poly (I:C) increases the activity of murine pannexin1 hemichannels. A**. Recording for 15 min of nuclear fluorescence intensity of DAPI in HeLa-mPanx1 cells in the presence of increasing concentrations of Poly (I:C). n = 3. **B**. DAPI uptake rate in response to increasing concentrations of Poly (I:C), normalized to the response obtained with 10 µg/mL. **C-E:** RT-qPCR analysis for IL-1β (A), TNF-α (B), and Panx1 (C) mRNA levels from RAW cells stimulated by 6 h with 10 µg/mL Poly (I:C) or Poly (I:C) plus 200 µM ^10^Panx1, a Panx1 HC inhibitor. n = 3**. F**. DAPI uptake rate in RAW or HeLa-Cx43 cells, in DCFS or Krebs alkaline solution to increase the activity of hemichannels or treated with ^10^Panx1, a blocker of Panx1 HCs (n = 3). **G**. ATP levels in the extracellular milieu of RAW cells in response to 30 min incubation with Poly (I:C) or Poly (I:C) plus ^10^Panx1. Alkaline Krebs solution (pH = 8.5) was used as a positive control (n = 3). Each point represents the mean ± SEM. one-way ANOVA followed by Tukey post hoc test. * p < 0.05; ** p < 0.01; *** p < 0.001.

Next, we wanted to evaluate the mechanism by which Panx1 would regulate the development of inflammation. To find out whether Poly (I:C) has a direct effect on the hemichannel activity, we used Cx45^-/-^ HeLa cells, which do not allow dye uptake or present hemichannel currents 24, and transfected them with mPanx1. In these cells, Poly (I:C) induced a rapid (<1 min) increase in DAPI uptake in a concentration-dependent manner (Figure 2A-B). As highlighted by the dye uptake rate values, maximum dye uptake was attained with 100 µg/mL Poly (I:C) (Figure 2B). To evaluate whether Panx1 HC activity participates in the inflammatory response, we used a cell line derived from mouse macrophages, namely RAW264.7. To this end, cells were incubated with 10 µg/mL Poly (I:C) or Poly (I:C) plus ^10^Panx1, a selective Panx1 HC inhibitor, for 6 h, after which mRNAs were extracted, and levels of messenger for TNF-α and IL-1β were evaluated. As expected, mRNA levels of both cytokines significantly increased in response to Poly (I:C), whereas coincubation with ^10^Panx1 prevented such a response, suggesting a relevant Panx1 HC dependence (Figure 2C-D). The Panx1 messenger was not significantly affected by any treatment (Figure 2E).

Because macrophages not only express Panx1, but Cx43 and Cx37 as well 31, 32, we evaluated whether Poly (I:C)-induced dye uptake is inhibited by preincubation with 10 µM carbenoxolone (CBX), a Cx and Panx1 HC blocker 23, 200 µM ^10^Panx1, a Panx1 HC blocker 6, or D4, a selective Cx HCs inhibitor 33 that does not affect Panx1 HCs 34. While the response to Poly (I:C) was completely prevented by CBX or ^10^Panx1, D4 did not alter the increase in DAPI uptake induced by 10 µg/mL Poly (I:C) (Figure 2F, white bars). While RAW cells responded to the divalent cation-free solution (DCFS), suggesting the activation of Cx HCs, Poly (I:C) showed no effects on HeLa-Cx43 cells, which presented a high response to DCFS that were inhibited with D4 (Figure 2F, black bars). Moreover, the acute application of Poly (I:C) did not affect DAPI uptake in Panx1^-/-^ RAW cells (Figure S3A-B). Concomitant with the increase in dye uptake, 10 µg/mL Poly(I:C) was sufficient to induce a significant increase in ATP release (2.00 ± 0.09 vs. 1.26 ± 0.07 nmol/mg protein, p = 0.001) in RAW cells, which was prevented by 200 µM ^10^Panx1 preincubation (1.41 ± 0.05) (Figure 2F). Alkaline pH (8.5) was used as a positive control for Panx1 HC activation 35, resulting in a 1.8-fold increase in ATP release compared to the control (Figure 2G). Interestingly, we found that acute application of Poly (I:C) not only increases the activity of murine Panx1 HCs, but also human Panx1 HCs (Figure S4A-B). Furthermore, the activation exerted by Poly (I:C) is much greater when tested in cells transfected with a C-terminally truncated form of hPanx1, which is associated with cell death by apoptosis 36. Although Poly (I:C) has been linked to the triggering of apoptosis in human cells 37, 38, the application of Poly (I:C) for 6 h was not sufficient to induce cleavage of hPanx1, nor did it favor the uptake of Etd^+^ in HeLa-hPanx1 cells (Figure S4C-E), which we have reported to be associated with cleaved hPanx1 HCs 39. Taken together, these results indicate that Poly (I:C) specifically increases the activity of both mouse and human Panx1 HCs, without bearing an effect over Cx43 HCs or other Cx HCs in RAW cells. Furthermore, at the times evaluated, Poly (I:C) did not promote C-terminal cleavage of hPanx1.

### Poly (I:C) induces a toll-like receptor 3-dependent increase in intracellular Ca^2+^

**Figure 3:**
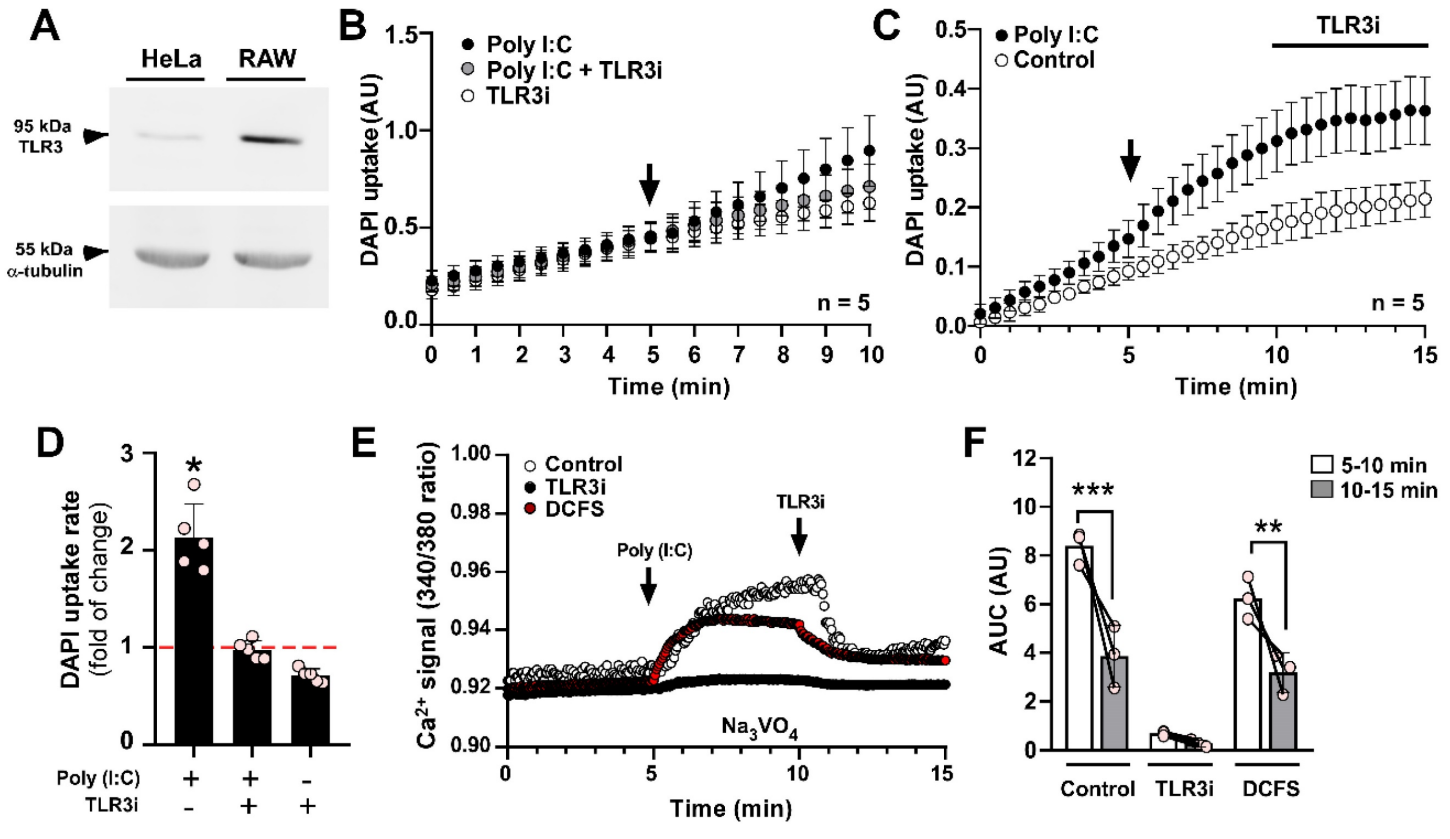
** Poly (I:C) induces a toll-like receptor 3-dependent increase in Ca^2+^ signal**. **A**. Western blot analysis of TLR3 protein in total homogenates of HeLa-mPanx1 and RAW cells. α-tubulin was used as a housekeeping protein. **B**. The inhibition of TLR3 signaling with 30 µM TLR3 inhibitor (TLR3i) prevented the Poly (I:C)-induced increase in Panx1 HC activity in HeLa-mPanx1 cells (n = 5). **C**. TLR3i blocks Panx1 HCs previously treated with 10 µg/mL Poly (I:C) (n = 5). **D**. Fold of change compared to baseline in DAPI uptake rate in response to Poly (I:C) or Poly (I:C) plus TLR3i. **E**. Representative records of intracellular Ca^2+^ signal (R340/380) in HeLa-mPanx1 cells treated with Poly (I:C) in Krebs solution (control), or divalent cation-free solution (DCFS) or in cells preincubated with TLR3 inhibitor (TLR3i). In all cases, the TLR3i effect was assayed after 5 min of Poly (I.C) treatment. **F**. Area under the curve from plot E (n = 3. Each point represents the mean ± SEM. * p < 0.05, ** p < 0.01, *** p < 0.001.

Since TLR3 is the main endogenous receptor for Poly (I:C) 40, we set out to verify if this receptor plays a role in the effect of Poly (I:C) on Panx1 HC activity. Despite HeLa cells having lower TLR3 expression compared to immune cells like RAW 264.7 (Figure 3A), we found that incubation of HeLa-mPanx1 cells with a TLR3 inhibitor (TLR3i) prevented the impact of Poly (I:C) on Panx1 HC activity (Figure 3B, D). Moreover, once these hemichannels became activated in response to Poly (I:C), the addition of TLR3i reduced the dye uptake rate to values comparable to baseline (Figure 3C). These results suggest that TLR3 is required for both enhancing and maintaining high hemichannel activity in response to Poly (I:C).

One of the main stimuli that activate Panx1 HCs is an increase in intracellular Ca^2+^ 22. To investigate whether Poly (I:C) induces a Ca^2+^ signal in HeLa cells transfected with mPanx1, we exposed the cells to 10 µg/mL Poly (I:C), and monitored Ca^2+^ levels using Fura-2 in the presence of 1 mM sodium orthovanadate, which is a Ca^2+^ pump blocker 41. In this condition, Poly (I:C) evoked a persistent increase in the Ca^2+^ signal (Figure 3E, control). It should be noted that full-length Panx1 HCs are not Ca^2+^ permeable, as we recently demonstrated 39. Therefore, this increase in Ca^2+^ signal is unlikely due to entry through Panx1 HCs in acute conditions. Importantly, pre-incubation with a TLR3 complex inhibitor (TLR3i) for 2 min before Poly (I:C) application almost entirely prevented the calcium transients (Figure 3E, black circles, and Figure 3F, TLR3i). Additionally, applying TLR3i after Poly (I:C) stimulation led to a ~55% reduction in the Ca^2+^ signal (AUC) (Figure 3E-F, control, 8.40 ± 0.39 vs. 3.85 ± 0.73 AU, p = 0.0001). To assess whether Ca^2+^ signal increase requires influx from the extracellular medium or release from intracellular reservoirs, we evaluated it in response to Poly (I:C), but in DCFS. Under these conditions, acute stimulation with Poly(I:C) increased Ca^2+^ signal to a slightly lower level than those obtained in the recording solution containing divalent cations (6.24 ± 0.50 vs. 8.40 ± 0.39 AU, p = 0.0427), suggesting a mixed response (Figure 3E-F, DCFS).

### Poly (I:C)-induced activation of pannexin1 hemichannels depends on P2X_7_R and reticular Ca^2+^ mobilization in RAW cells

**Figure 4:**
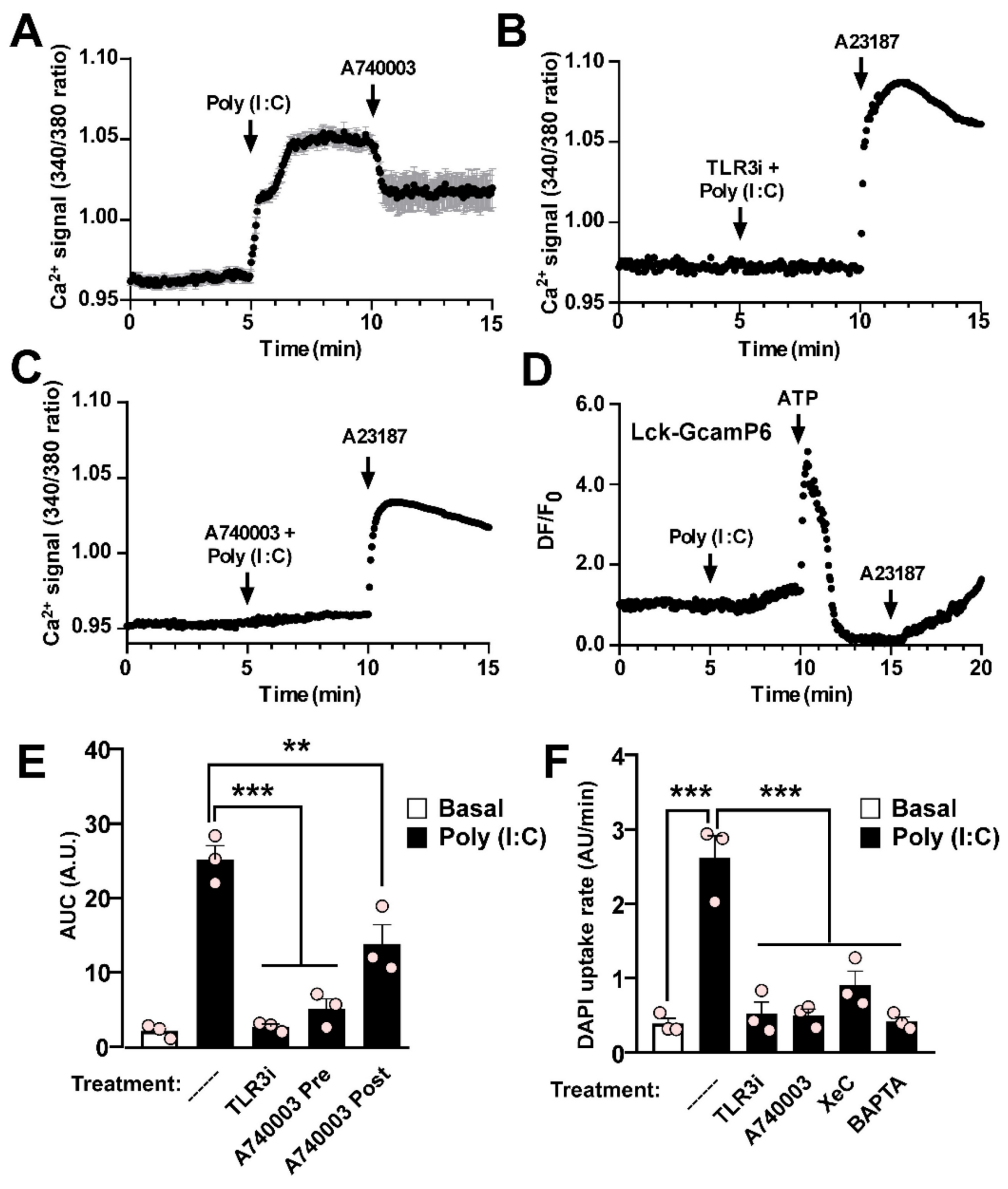
** Poly (I:C)-induced activation of pannexin1 hemichannel depends on toll-like receptor 3, Ca^2+^ mobilization, and P2X_7_R activity in RAW cells. A-C.** Representative records of Fura-2 fluorescence in RAW cells stimulated with Poly (I:C) (A) or assayed in combination with 30 µM TLR3i (B), or 20 µM A740003 (C). In B and C, 5 µM A23187 Ca^2+^ ionophore was used for demonstrating membrane integrity. Cells were loaded with Fura-2 and recording was made in the presence of 1 mM Na_3_VO_4_ (n = 3). **D**. RAW cells were transfected with Lck-GcamP6 and 24 h later GcamP6 fluorescence was evaluated in response to Poly (I:C) or 0.5 mM ATP (n = 3). **E**. Area under the curve from A-C. **F**. DAPI uptake rates of RAW cells assayed in response to 10 µg/mL Poly (I:C) or preincubated with 30 µM TLR3i, 20 µM A740003, 5 µM XeC or 10 µM BAPTA ** p < 0.01; *** p < 0.001.

As observed in HeLa-mPanx1 cells, the Ca^2+^ signal of RAW cells significantly increased in response to Poly (I:C) (Figure 4A), and this response was entirely prevented by preincubation with TLR3i (Figure 4B). The fast increase in Ca^2+^ signal suggests the involvement of a P2XR activation that favors a Ca^2+^ influx. Since P2X_7_R plays a relevant role in the inflammatory responses of macrophages, we next evaluated its participation in the intracellular Ca^2+^ increase described above. We found that preincubation with A740003, a selective P2X_7_R blocker 42, almost completely prevents the response to Poly (I:C) in RAW cells (Figure 4C). Interestingly, the application of A740003 after Poly (I:C) reduced the response to Poly (I:C) by approximately half (Figure 4A). These findings are consistent with the partial reduction in Ca^2+^ signal observed in HeLa cells upon exposure to DCFS, suggesting that Ca^2+^ influx through P2X_7_R may play an important role in a second phase of the response. Furthermore, in agreement with the notion that Poly (I:C) first evokes Ca^2+^ mobilization from reticular sources, its application did not significantly change the fluorescence intensity of the sub-membranous Ca^2+^ probe Lck-GCamP6, despite a slight secondary increase after 2 min of treatment (Figure 4D). However, 0.5 mM ATP generated a large increase in the fluorescence detected with this probe, which may have been associated with Ca^2+^ influx by P2XRs. The area under the curve (AUC) of changes in Ca^2+^ signal (Figure 4E) was accompanied by analogous changes in dye uptake in RAW cells, where pre-incubation with TLR3i or A740003 led to a significant reduction in Panx1 HC activity in response to Poly (I:C), while the response to alkaline solution persisted (Figure 4F and Figure S5A). Moreover, preincubation of cells with either xestospongin C (XeC), an IP_3_R antagonist, or BAPTA-AM, a Ca^2+^ chelator, avoided the increase in Panx1 HC activity induced by Poly (I:C) (Figure 4F and Figure S5B), indicating that Ca^2+^ mobilization from intracellular reservoirs participates in elevating Panx1 HC activity in response to Poly (I:C).

### Poly (I:C) induces Panx1-P2X_7_R interaction

**Figure 5:**
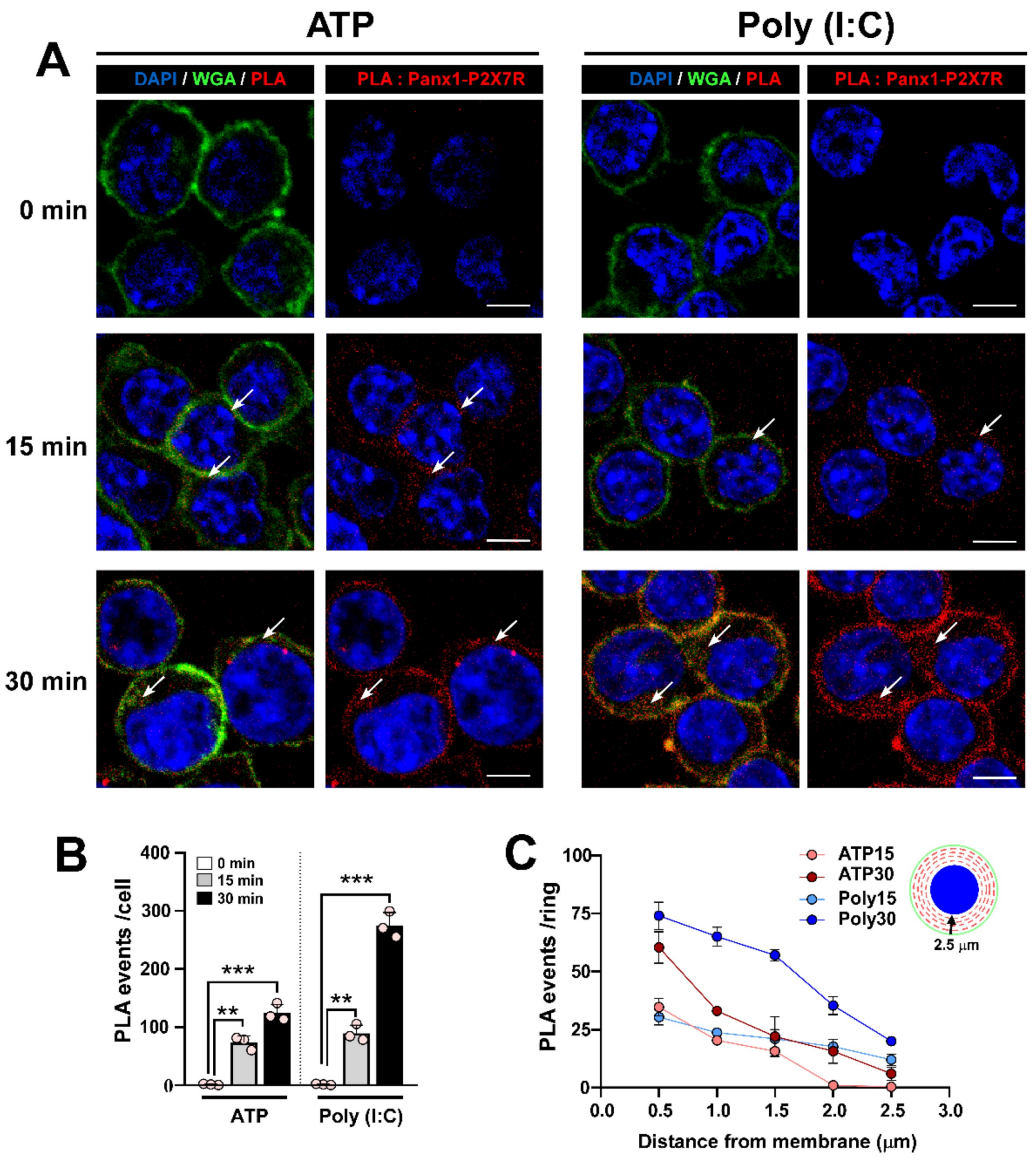
** Poly (I:C) favors the interaction of pannexin1 with P2X_7_R and complex internalization. A.** Confocal images of RAW cells incubated for 15 or 30 min with 0.5 mM ATP or 10 µg/mL Poly (I:C) and then processed by proximity ligation assay (PLA). Each PLA event (red spot) represents a site where Panx1 interacts with P2X_7_R. Alexa fluor 488-conjugated WGA and DAPI were used as a membrane and nucleus marker, respectively. The arrows indicate regions of the cell where PLA events are preferentially localized. Representative images from 3 independent experiments. Scale bar = 10 µm (n = 4). **B**. Total number of PLA events in each condition. **C**. For each cell, concentric rings were drawn from membrane to nucleus each 0.5 µm, and PLA event numbers were quantified in each ring. Each point represents the mean ± SEM for a condition from an independent experiment. ** p < 0.01, *** p < 0.001.

Previous studies have shown that a 15-minute application of extracellular ATP promotes interaction and internalization of the Panx1-P2X_7_R protein pair in N2a cells 43. Given that Poly (I:C) enhances ATP release through Panx1 HCs and relies on P2X_7_R for the increase in intracellular free-Ca^2+^ level induced by Poly (I:C), we next studied whether Poly (I:C) modifies the interaction or subcellular localization of this protein pair in RAW cells. To this end, we studied both parameters (interaction and internalization) using proximity ligation assay (PLA) and confocal microscopy. Firstly, we observed that 0.5 mM ATP application favored a time-dependent increase in PLA events (red spots), ranging from 0 to 124 events per cell (Figure 5A-B). In cells treated with 10 µg/mL Poly (I:C), a similar time-dependence trend was observed, but with a significantly greater number of PLA events compared to ATP treatment for 30 min (274.7 ± 12.9 vs. 124.0 ± 8.6, p = 0.0006). To determine whether each treatment affected the membrane localization of the protein pair, we determined that RAW cells have an average diameter of 12 µm, with approximately 2.5 µm from the membrane (WGA positive) to the nucleus (DAPI positive). In this way, due to the circular shape of these cells, we divided the cytoplasm into concentric rings every 0.5 µm, and quantified PLA events within each ring, as represented in the inset diagram (Figure 5C, inset). We found that ATP application for 15 min favored Panx1-P2X_7_R interaction mainly in rings 1-3 (i.e., the first 1.5 µm from the membrane) (Figure 5C). After 30 min of ATP treatment, a greater proportion of events occurred in the first ring, mostly corresponding to membrane or submembrane localization. However, the rest of the cytoplasm showed similar Panx1-P2X_7_R interactions as those observed from ATP treatment for 15 min. Poly (I:C) application for 15 min promoted a similar distribution of PLA events compared to ATP. However, following 30 min of Poly (I:C) incubation, interaction events were concentrated at high and similar levels in rings 1-3 (Figure 5C). Additionally, when the proteins were immunoprecipitated with the anti-P2X_7_R antibody, an intense band corresponding to Panx1 was detected in the samples from ATP and Poly (I:C) treatment, reinforcing that both proteins interact under these conditions (Figure S6). Similar results were observed by immunodetection of each protein, increasing cytoplasmic localization of Panx1 and P2X_7_R in response to Poly (I:C) (Figure S7A-B). Furthermore, interaction and internalization were inhibited by preincubation with bafilomycin A1 (BafA1) or apyrase, suggesting that endocytosis and extracellular ATP, respectively, are necessary for Panx1-P2X_7_R complex internalization (Figure S7C-D). These results suggest that Poly (I:C) first promotes greater interaction between Panx1 HC and P2X_7_R in the membrane, but after 30 min endocytosis of the complex was favored.

### Poly (I:C)-induced pannexin1 hemichannel activity relies on Ca^2+^/calmodulin-dependent protein kinase II

**Figure 6:**
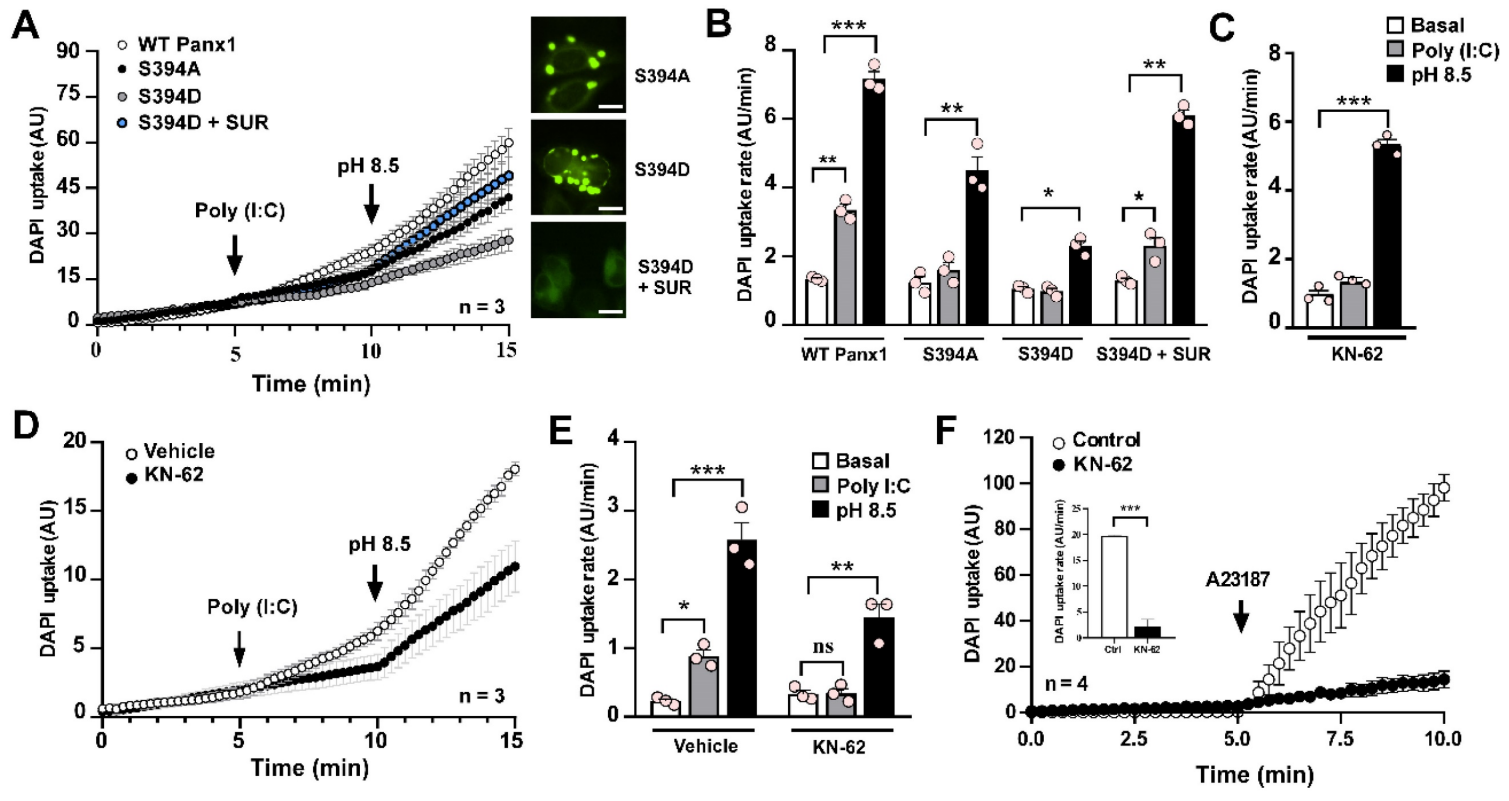
**The Poly (I:C)-induced activity of pannexin1 hemichannel depends on CaMKII activity**. **A-B**. rPanx1 mutants S394A and S394D (target site for CaMKII) were evaluated for Poly (I:C) susceptibility, and the dye uptake rates were calculated (B). For mutant S394D, an additional condition was run in which the cells were incubated with 100 µM suramin immediately post-transfection. Scale bar = 20 µm (n = 3). **C**. DAPI uptake rates from HeLa-rPanx1 cells preincubated with 10 µM KN-62 and then treated with 10 µg/mL Poly (I:C) for 5 min. **D**. DAPI fluorescence intensity in HeLa-hPanx1 cells preincubated for 30 min with 10 µM KN-62 followed by Poly (I:C) application. As a control, other cells were pre-incubated with 0.1% DMSO (vehicle for KN-62). **E**. Dye uptake rate calculated from C, considering basal conditions (0-5 min), stimulation with Poly (I:C) (5-10 min), and alkaline Krebs (pH = 8.5) application (10-15 min) (n = 3). **F**. DAPI uptake in cells treated with the Ca^2+^ ionophore A23187 (10 µM) alone or after 30 min pre-incubation with KN-62 followed by application of the Ca^2+^ ionophore. The inset shows DAPI uptake rates for each condition in response to Poly (I:C). Alkaline Krebs buffer was applied for 5 minutes at the end of some recordings to demonstrate the presence of hemichannels in the membrane (n = 4). Each point represents the mean ± SEM. One-way ANOVA followed by Tukey post hoc test. * p < 0.05; ** p < 0.01; *** p < 0.001.

We previously reported that mechanical stretch-induced increases in rPanx1 HC activity are triggered by Ca^2+^/calmodulin-dependent protein kinase II (CaMKII) activity 44. Specifically, CaMKII phosphorylates the Ser394 residue, enhancing rPanx1 HC activity upon mechanical stimulation. To test whether Poly (I:C) affects Panx1 HC activity through this post-translational modification, we transfected HeLa cells with WT rPanx1-EGFP, or the mutants S394A and S394D. In cells transfected with WT Panx1, we recorded a 2.7-fold increase in DAPI uptake rate in response to Poly (I:C) (3.35 ± 0.16 vs. 1.33 ± 0.04 AU/min, p = 0.001). Exposure to alkaline (pH: 8.5) saline solution further amplified this response (7.17 ± 0.21 AU/min, p = 0.0003 compared to basal) (Figure 6A-B). Interestingly, Panx1 mutants in which this phosphorylation target was abolished (S394A) did not respond to Poly (I:C) (1.4 ± 0.1 vs. 1.8 ± 0.2 AU/min. p = 0.9606), demonstrating the role of this post-translational modification in this response. However, the S394A mutant retained its response to alkaline pH (4.50 ± 0.40 AU/min), indicating HC presence in the cell membrane that can be activated through a different gating mechanism. Based on our previous report using a phosphomimic mutant of Panx1 (S394D) 23, it was mostly observed to be organized intracellularly in a vesicular pattern, which is consistent with the null response to Poly (I:C) (0.98 ± 0.08 vs. 1.05 ± 0.06), with minimal response to alkaline pH (2.29 ± 0.15, p = 0.0136 compared to basal) (Figure 6A-B). Surprisingly, in S394D-Panx1-transfected cells treated with suramin (a P2 receptor antagonist 45) for 12 h, rPanx1 exhibited a membrane pattern similar to WT isoform. Under these conditions, responses to Poly (I:C) and alkaline pH were enhanced (2.30 ± 0.24 AU/min and 6.10 ± 0.16, AU/min, respectively), suggesting that the response to Poly (I:C) requires greater membrane distribution of Panx1. This distribution, in turn, depends on the phosphorylation state of Ser394 and P2R activity. Interestingly, pharmacological inhibition of CaMKII with KN-62 46 in HeLa cells expressing WT rPanx1 generated the same outcome observed using the S394A mutant: Loss of the Poly (I:C) response while retaining the alkaline pH response (Figure 6C). Notably, the inhibition of CaMKII, as well as the inhibition of Panx1 HC or P2X_7_R, prevented the interaction and internalization observed in response to Poly (I:C) (Figure S8), reinforcing the hypothesis that prolonged activation of the CaMKII/Panx1/P2X_7_R pathway mediates protein complex endocytosis.

To determine whether CaMKII modulates Panx1 HC activity through Ser394 in response to Poly (I:C), we studied the effect of KN-62 in HeLa cells transfected with human Panx1 (hPanx1), which lacks Ser394. Under CaMKII inhibition, Poly (I:C) did not alter the activity of hPanx1 HCs compared to the basal condition (0.34 ± 0.07 vs. 0.33 ± 0.05 AU/min) (Figure 6D-E). However, alkaline pH-induced activity remained intact (1.45 ± 0.19 AU/min, p = 0.0011 compared to baseline), confirming the presence of hPanx1 HCs in the cell membrane (Figure 6D-E). In HeLa-hPanx1 cells pre-incubated with vehicle (0.1% DMSO), Poly (I:C) significantly increased dye uptake rates (0.88 ± 0.23 vs. 0.23 ± 0.03 AU/min, p = 0.0463), which further increased upon exposure to extracellular alkaline pH (2.58 ± 0.25, p = 0.0001 compared to baseline) (Figure 6E, vehicle). Consistent with the concept that elevated cytoplasmic Ca^2+^ activates CaMKII, which subsequently enhances Panx1 HC activity, KN-62 drastically reduced the increase in hPanx1 HC activity induced by A23187, a Ca^2+^ ionophore 47, by approximately 89% (19.72 ± 0.12 vs. 2.19 ± 0.68 AU/min, p = 0.0003) (Figure 6F and inset). Thus, the enhancement of hPanx1 HC activity promoted by a rise in cytoplasmic Ca^2+^ depends on CaMKII activity. Because hPanx1 lacks Ser394 residue, but CaMKII dependency persists, the contribution of an alternative phosphorylation site not yet identified or the involvement of an indirect signaling pathway cannot be ruled out.

### Protein kinase A activation abolishes Poly (I:C)-induced pannexin1 hemichannel activity

**Figure 7:**
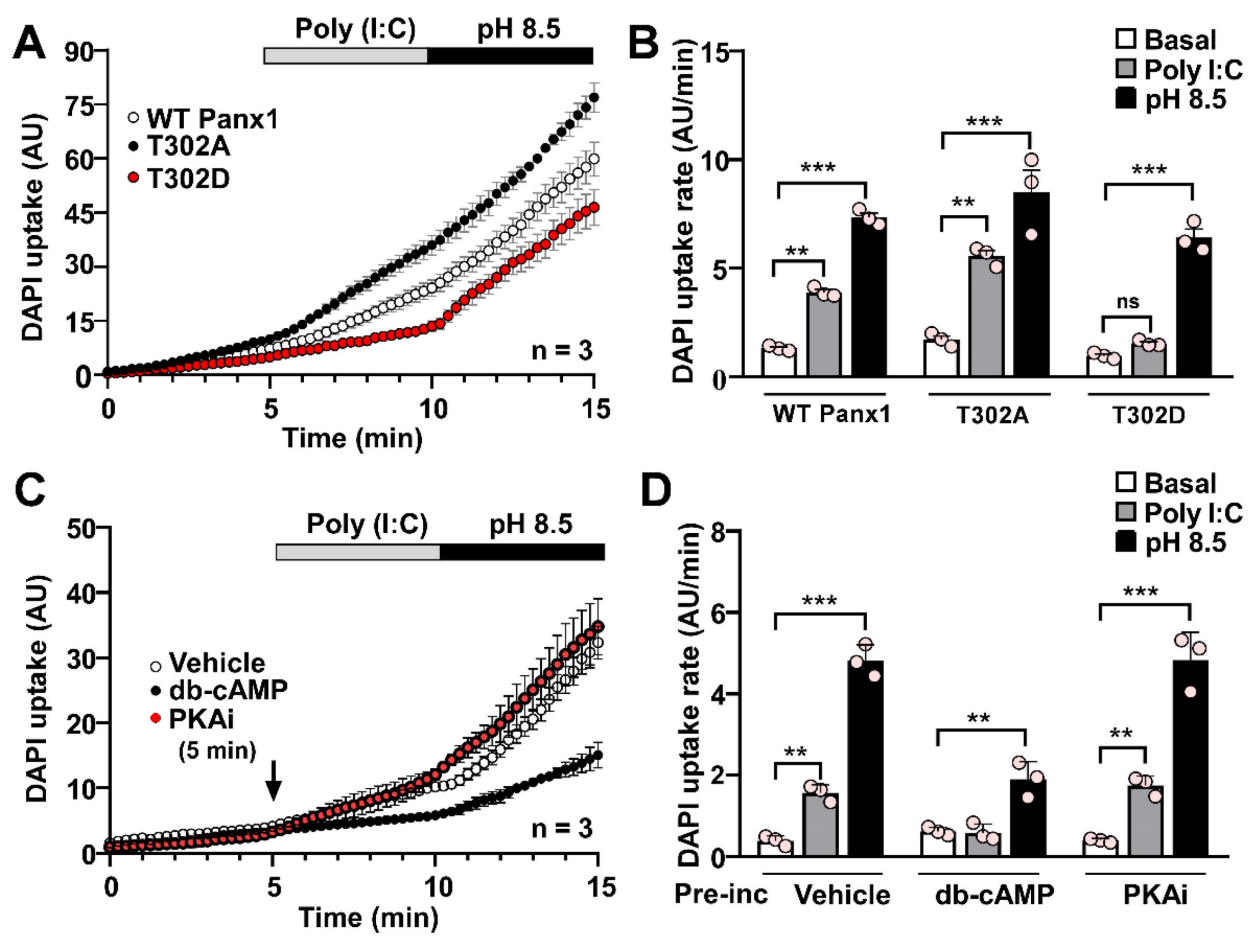
** PKA-dependent phosphorylation prevents the Poly (I:C)-induced increase in pannexin1 hemichannel activity**. **A**. DAPI uptake of HeLa parental cells (Cx45^-/-^, Panx1^-/-^) after 12 h of transfection with rPanx1-T302A or rPanx1-T302D (target site for PKA) (n = 3). **B**. Dye uptake rate from A for every condition and Panx1 mutant. **C**. Dye uptake was recorded in HeLa-hPanx1-mCherry cells pre-incubated or not with either 500 µM db-cAMP or 20 µM PKAi by 5 min before 10 µg /mL Poly (I:C) application. As a control, other cells were pre-incubated with PBS (vehicle). In each condition, cells were stimulated with Krebs solution pH 8.5 to demonstrate the presence of Panx1 HCs in the cell membrane (n = 3). **D**. Dye uptake rate calculated from C, considering basal conditions (0-5 min), stimulation with Poly (I:C) (5-10 min), and application of alkaline Krebs solution (10-15 min). Each point represents the mean ± SEM. One-way ANOVA followed by Tukey post hoc test. ** p < 0.01; *** p < 0.001.

Both murine and human Panx1 contain PKA target sites likely involved in regulating HC activity since evidence suggests that PKA activation prevents the response to mechanical stimulation on rPanx1-expressing cells 44. In HeLa cells transfected with an rPanx1 mutant, where a PKA phosphorylation site was replaced by an alanine residue (S302A), DAPI uptake rate was significantly higher than basal conditions in response to Poly (I:C) (5.64 ± 0.19 AU/min vs. 1.71 ± 0.17 AU/min, p = 0.0015). Furthermore, DAPI uptake was slightly higher (not statistically significant) than that elicited by WT rPanx1 HCs in response to Poly (I:C) (3.90 ± 0.13, p = 0.09) (Figure 7A-B). Conversely, the activity of hemichannels formed by the phosphomimetic mutant (S302D) was not affected by Poly (I:C), remaining comparable to that of baseline (1.55 ± 0.08 vs. 0.97 ± 0.08 AU/min) (Figure 7A-B). In all cases, the application of alkaline pH led to at least a 6-fold increase in rPanx1 HC activity, confirming the presence of hemichannels in the cell membrane. To corroborate that hPanx1 HC regulation by Poly (I:C) depends on PKA activity, we used db-cAMP, a membrane-permeant PKA agonist 48. In HeLa-hPanx1 cells preincubated with vehicle (PBS), Poly (I:C) or alkaline pH evoked a 4-fold and 12-fold increase in dye uptake rate, respectively (Figure 7C-D, vehicle). Interestingly, a 5-min pre-incubation with 500 µM db-cAMP was sufficient to prevent the Poly (I:C) effect on hPanx1 HC activity (0.59 ± 0.12 vs. 0.62 ± 0.06 AU/min), whereas alkaline pH still evoked a 4-fold increase in DAPI uptake rate, confirming the presence of hPanx1 HCs in the membrane (Figure 7D). Moreover, cells simultaneously treated with a PKA inhibitor (PKAi) and Poly(I:C) showed a significant increase in dye uptake rate (1.75 ± 0.13 vs. 0.40 ± 0.03 AU/min, p = 0.0022), comparable to that of cells preincubated with vehicle (Figure 7D). In summary, although the response to alkaline pH was retained, PKA-dependent S302 phosphorylation not only slows down the response to mechanical stress, as reported for rPanx1 expressing cells 44, but also modulates the impact of other stimuli such as Poly (I:C) on murine and human Panx1 HC activity.

### Poly (I:C) stimulation causes a Ca^2+^/calmodulin-dependent protein kinase II-dependent increase in proinflammatory cytokines

**Figure 8:**
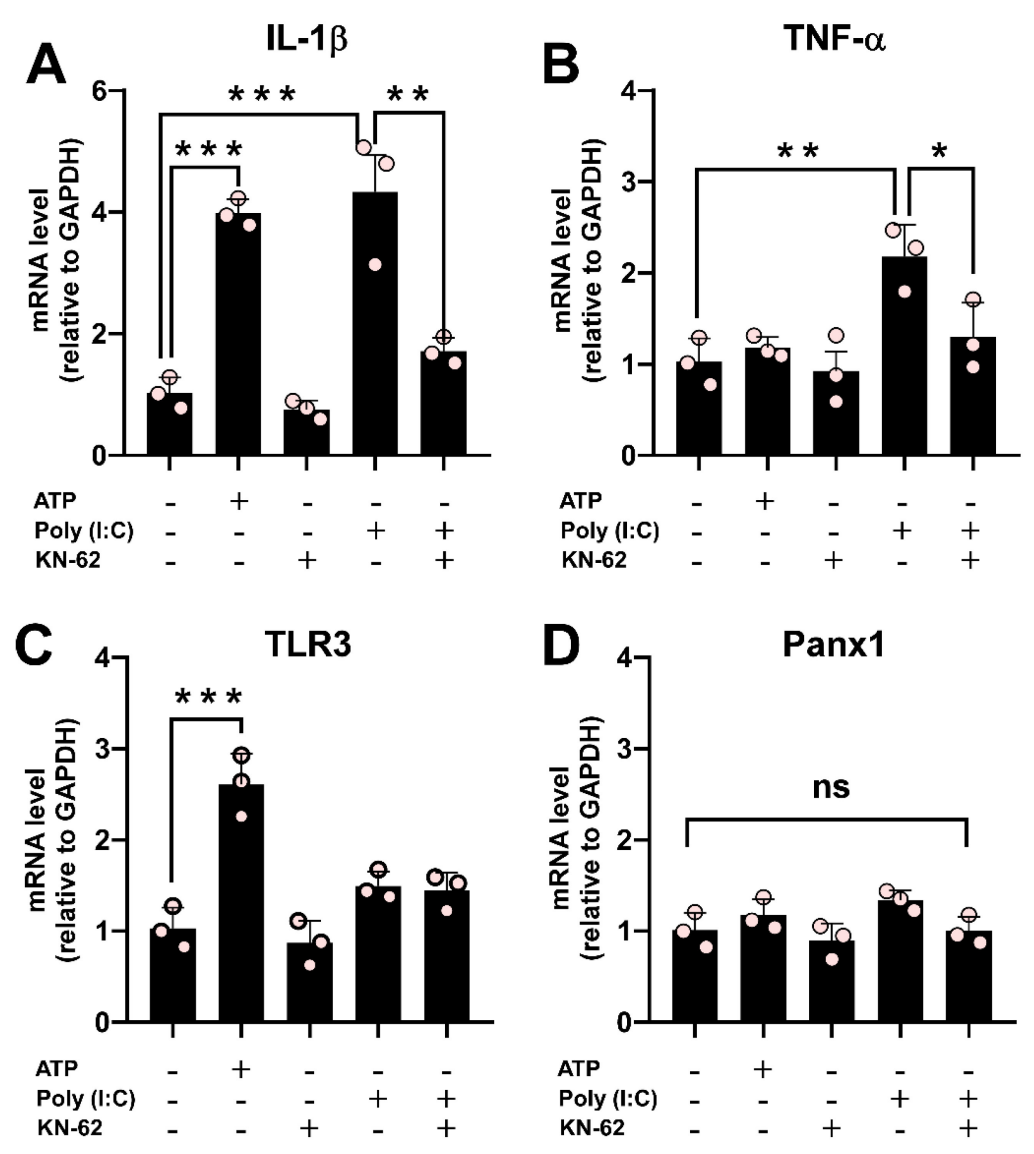
** The Poly (I:C)-induced proinflammatory response depends on the activity of CaMKII in RAW cells**. Raw cells (3.3 x10^4^ cells/cm^2^) were seeded in 35 mm dishes and 24 h later were incubated for 6 h with i) 10 µg/mL Poly (I:C), ii) 0.5 mM ATP, iii) 10 µM KN-62 or iv) mixtures of Poly (I:C) and KN-62. RNA was extracted from each plate and processed for reverse transcription. Using each cDNA as a template, the mRNA levels of IL-1β (**A**), TNF-α (**B**)**,** TLR3 (**C**), and Panx1 (**D**) were evaluated by qPCR. GAPDH was used as the reference gene and each value was normalized to the control condition using the 2^-ΔΔCt^ method (n = 3). Each point represents the mean ± SEM. one-way ANOVA followed by Tukey post hoc test. * p < 0.05; ** p < 0.01, *** p < 0.01.

Our results have demonstrated that the effect of Poly (I:C) on Panx1 HC activity depends on CaMKII. To evaluate the involvement of the CaMKII / Panx1 axis on inflammation triggering, we next co-incubated RAW cells with Poly (I:C) and KN-62 for 6 h. While KN-62 application did not promote changes, its preincubation prevented the upregulation of IL-1β (1.71 ± 0.13 vs. 4.33 ± 0.60, p = 0.0073) and TNF-α (1.30 ± 0.22 vs. 2.18 ± 0.20, p = 0.0355) mRNA levels induced by Poly(I:C). This strongly suggests that the activation of the CaMKII/Panx1 axis is critical for evoking an inflammatory response (Figure 8A-B). ATP, known to induce IL-1β release in immune cells 6, 49, significantly upregulated IL-1β (3.99 ± 0.13 folds, p = 0.0003) and TLR3 (2.61 ± 0.19 folds, p = 0.0004) mRNA levels compared to control conditions (1.00). The mRNA level of TLR3 remained unchanged in the other conditions tested (Figure 8C). Furthermore, Panx1 mRNA levels were unaffected across all conditions (Figure 8D). Taken together, our results highlight the critical role of the TLR3/Ca^2+^/CaMKII/Panx1 HCs axis in mediating an inflammatory response triggered by TNF-α or IL-1β in response to viral patterns.

## Discussion

Our study found that Poly (I:C) leads to an increase in Panx1 HC activity, in both a simple model that only expresses this non-selective channel, but also in more complex natural systems with endogenous Panx1 expression. In reconstituted exogenous expression systems, such as HeLa-Panx1 cells, Poly (I:C) enhances Panx1 HC activity via TLR3, intracellular Ca^2+^, and CaMKII. In RAW cells with endogenous Panx1 expression, this signaling triggered proinflammatory cytokine expression in response to Poly (I:C). In addition, Panx1 expression was pivotal in evoking an inflammatory response to Poly (I:C) injection in mice, both in abdominal muscle (close to the injection site) and peritoneal macrophages. There are several lines of evidence demonstrating that an increase in Panx1 HC activity mediates the release of ATP (danger signal), which enhances inflammatory responses implicated in inflammasome activation and leukocyte recruitment. In this context, Panx1 HCs play a role in the production and secretion of proinflammatory cytokines (such as IL-1β and IL-6) by endothelial cells and other cell types 6, 50-52. However, although the link between HC activity and immune responses is well-established, the specific interplay between viral molecular patterns, Panx1 HC activity, and inflammation was previously unexplored.

We demonstrated that the increase in Panx1 HC activity in response to Poly (I:C) relies on TLR3 and Ca^2+^ mobilization from endoplasmic reticulum stores. This finding aligns with previous reports suggesting that TLR3 activation may evoke IP_3_R activation in human mesenchymal stem cells 20. Considering that the increase in intracellular Ca^2+^ signal persists (albeit briefly) in the absence of extracellular Ca^2+^ in response to Poly (I:C), and that incubation with a P2X_7_R antagonist partially reverses the effect of Poly (I:C), it is plausible to hypothesize that the response to Poly (I:C) is exerted in two phases: i) TLR3 signaling and reticular Ca^2+^ mobilization leads to the initial increase in Panx1 HC activity and release of ATP; ii) extracellular Ca^2+^ entry, likely mediated by P2XR signaling, which further enhances the response. Interestingly, our results revealed that preincubation of RAW cells with A740003 (a P2X_7_R inhibitor) prevents both an increase in dye uptake and intracellular Ca^2+^ signal in response to Poly (I:C), emphasizing the importance of purinergic signaling enhancing the Poly (I:C) response. It should be noted that proinflammatory cytokines like TNF-α have been reported to enhance ATP release mediated by Panx1 HCs in various cell models 14, 53. Thus, this autocrine mechanism could contribute to the observed effects once these cytokines are released in our model.

In an eventual feedforward purinergic cycle, where Ca^2+^ entry and increased Panx1 HC activity leads to further ATP release and P2 receptor activation, regulatory mechanisms are likely to be in place to prevent cell death from this cascade. In line with this hypothesis, our results showed that upon long exposure to Poly (I:C) or ATP (30 min), Panx1 HCs interacted with P2X_7_Rs, acquiring a more intracellular localization. Similar results have been reported for this protein pair in N2a cells in response to ATP, albeit with a high interaction before ATP application 43. Additionally, Jiang and Chen demonstrated that ATP and glutamate greatly enhance dynamin-independent endocytosis by elevating intracellular free-Ca^2+^ level in astrocytes 54. It is necessary to highlight that in RAW cells, exposure to Poly (I:C) for 6 h induced a significant upregulation of both TNF-α and IL-1β. This suggests that even if the Panx1 HC-P2X_7_R complex remained internalized after 6 h of stimulation with Poly (I:C) (not evaluated), the inflammatory response persisted. This could be attributed to either a robust initial inflammatory response with downstream effects persisting and/or a short half-life of the stimulus, causing the protein pair to return to the membrane. Supporting the latter possibility, seminal evidence indicates that the half-life of Poly (I:C) is less than 30 min in serum 55. Future studies could shed light on the dynamics of this phenomenon in cell cultures, and whether Panx1 HC activity diminishes following internalization.

To our knowledge, the precise mechanism by which an increase in intracellular Ca^2+^ signal causes activation of hPanx1 HCs remains elusive. Unlike conventional Ca^2+^-binding domains (such as EF-hand or Ca^2+^ bowl), Panx1 HCs lack such features 56. However, we propose that a Ca^2+^/CaMKII interaction may instead explain the Ca^2+^-mediated activation of Panx1 HCs. Previously, we reported that CaMKII-dependent phosphorylation at Ser394 enhances Panx1 susceptibility to mechanical stress 23. Here, we demonstrated that this same post-translational modification on Panx1 also strongly regulates hemichannel susceptibility to Poly (I:C). Even though the hypothesis was that phosphorylation by CaMKII would enhance Panx1 HC activity, the phosphomimetic mutant S394D presented an endosomal pattern, as previously reported 23, which prevented a normal response to Poly (I:C), and a partial one to alkaline solution. I should be noted that this lack of response was reverted by preincubation with suramin. While further experimental clarification is needed, this regulation aligns with the notion that robust purinergic signaling, such as that induced by prolonged exposure to Poly (I:C), and subsequent high extracellular ATP concentration leads to CaMKII-dependent endocytosis of the Panx1 HC-P2X_7_R complex, as discussed earlier.

The regulation of Panx1 HC activity by CaMKII was also demonstrated with the antagonist KN-62. Surprisingly, the inhibition of CaMKII also prevented Poly (I:C)-mediated activation in hPanx1 HCs, which do not possess the Ser394 residue. Using phosphorylation prediction servers (GPS 6.0 and NetPhos-3.1), no potential sites of phosphorylation by CaMKII were found in the human paralog. Consequently, an indirect mechanism cannot be ruled out. Regardless of the specific mechanism, in addition to preventing the upregulation of Panx1 HC activity by Poly (I:C), the application of KN-62 also inhibited the activation exerted by an ionophore-mediated massive increase in intracellular Ca^2+^ signal, suggesting that Ca^2+^ effects over Panx1 HCs largely depend on CaMKII activity. The functional relevance of phosphorylation mediated by CaMKII is such that our results showed that inhibiting this kinase in macrophages prevents the upregulation of proinflammatory cytokines in response to Poly (I:C). Although the association with Panx1 has not been previously addressed, the involvement of Ca^2+^/ CaMKII in inflammation is in line with reports where chelation of Ca^2+^ inhibits IL-1β processing and release in murine macrophages 57. Additionally, small molecule inhibitors have demonstrated that both Ca^2+^ and calmodulin are required for nigericin-induced IL-1β secretion in THP-1 cells and primary human monocytes 58. Furthermore, activation of TLR3, TLR4, or TLR9 leads to CaMKII activation and production of IL-6, TNF-α, and IFN-α/β in macrophages 59. These observations open the doors to focusing on this axis as a therapeutic target to modulate the development of inflammation, especially in the context of viral infections.

In addition to the regulation exerted by CaMKII, our results highlight the significant role of PKA activity in the response of Panx1 HCs to Poly (I:C). Particularly, T302D phosphomimetic mutation and exposure to PKA activator db-cAMP were sufficient to counteract the Poly(I:C)-mediated effect. This is consistent with our previous findings where PKA phosphorylation at Thr302 or Ser328 of Panx1 prevents HC activation upon mechanical stretch. An intriguing question pertains to the mechanisms involved in reversing Panx1 phosphorylation exerted by CaMKII or PKA. Since calcineurin is a Ca^2+^-dependent phosphatase stimulated by calmodulin, this enzyme could dephosphorylate PKA target sites on Panx1 upon CaMKII activation, explaining the opposite regulation exerted between CaMKII and PKA. Further studies will provide clarity on these intriguing aspects.

Our results strongly suggest that an increase in Panx1 HC activity plays a fundamental role in the initial phase of viral infections, particularly when dsRNAs are present. It is important to highlight that Panx1 expression was also crucial for Poly (I:C) internalization, suggesting that managing the activity of these hemichannels could help to reduce viral load. In concordance, Panx1 HCs have been implicated in several viral infections, such as HIV infection of human primary cells. HIV induces an early and transient increase in Panx1 HCs, which was observed in peripheral blood mononuclear cells (PBMCs) and CD4^+^ T lymphocytes 18. Regarding respiratory virus infections, inflammation affects various tissues throughout the body beyond the lungs. For instance, in severe cases of SARS-CoV-2 infection, intense vascular inflammation is present, which is often the cause of major complications. Due to the widespread Panx1 expression in vertebrates, Panx1 HCs represent a potential target for reducing the inflammatory burden and mitigating the damaging effects of the cytokine storm in COVID-19 patients. Recent research indicates that the SARS-CoV-2 Spike protein increases Panx1 HC activity in human lung primary epithelial cells, facilitating virus entry and ATP release 61. However, beyond Panx1 HC, it remains to be determined whether other members of the proposed signaling pathway are involved in prevalent viral infections, such as SARS-CoV-2 infection. Future studies will help us determine whether the same signaling pathway is activated in response to whole RNA viruses and is not limited to a viral pattern such as Poly (I:C).

The eventual therapeutic management of signaling mediated by TLR3 / CaMKII / Panx1 HCs requires determining the concentrations that could induce a protective rather than a detrimental response. It should be noted that TLR3 also plays an important role in antiviral defenses by triggering a potent immune response against dsRNAs produced during viral infection. While the role of TLR3 in protection against viral infections may vary among viruses, its detrimental contribution to viral pathogenesis is predominant according to studies in mice and humans, especially after massive inflammatory responses to viruses 63. Regarding CaMKII, its inhibition has been mostly related to beneficial effects. Still, some adverse side effects cannot be ignored since this kinase is believed to regulate neurotransmission and synaptic plasticity in response to calcium signaling produced by neuronal activity. For instance, CaMKII inhibition in cultured cortical neurons induces neurotoxicity by disrupting Ca^2+^ and glutamate homeostasis, increasing excitability, and inducing apoptosis 64.

Taking together, our results demonstrate the pivotal role of Panx1 HCs in response to viral patterns. Particularly, Panx1 HC activity increases in response to Poly (I:C), relying on Ca^2+^/CaMKII activation. When extracellular ATP accumulates, a second phase is activated, allowing Ca^2+^ influx and further enhancing CaMKII / Panx1 HC axis activity. As this signaling intensifies, the Panx1 HC-P2X_7_R complex is endocytosed, likely limiting runaway signaling. Given that the inflammatory response against Poly (I:C) in macrophages depends on the activity of this signaling cascade, we believe that it emerges as a strong therapeutic target for managing inflammatory crises during viral infections.

## Supplementary Material

Supplementary figures.

## Abbreviations

ATP: adenosine triphosphate
BafA1: bafilomycin A1
BAPTA-AM: 1,2-Bis(2-aminophenoxy)ethane-N,N,N′,N′-tetraacetic acid tetrakis(acetoxymethyl ester)
BSA: bovine serum albumin
CaMKII: calcium/calmodulin-dependent protein kinase II
CBX: carbenoxolone
CRISPR: clustered regularly interspaced short palindromic repeats
DAPI: 4′,6-diamidino-2-fenilindol
db-cAMP: dibutyryl cyclic adenosine monophosphate
DCFS: divalent cation free solution
ECL: enhanced chemiluminescence
EGFP: enhanced green fluorescent protein
Etd+: ethidium bromide
Fura-2 AM: 1-[2-(5-Carboxyoxazol-2-yl)-6-aminobenzofuran-5-oxy]-2-(2ʹ-amino-5ʹ-methylphenoxy)-ethane-N,N,NʹNʹ-tetraacetic acid pentaacetoxymethyl ester
HC: hemichannel
HIV: human immunodeficiency virus
Iba1: ionized calcium-binding adapter molecule 1
IL-1β: interleukin 1β
lck-GCaMP3: lck fused to GFP-calmodulin-M13
MAPK: mitogen-activated protein kinase
mRNA: messenger ribonucleic acid
M-PER: mammalian protein extraction reagent
Panx1: pannexin1
PBS: phosphate saline buffer
PKA: protein kinase A
PM: peritoneal macrophage
P2X_7_R: P2X_7_ receptor
RT-qPCR: reverse transcription quantitative polymerases chain reaction
SARS-CoV-2: severe acute respiratory syndrome coronavirus 2
SDS-PAGE: sodium dodecyl sulfate polyacrylamide gel electrophoresis
TLR3: toll-like receptor 3
TNF-α: tumor necrosis factor α
XeC: xestonpongin C
