# Supplementary material for: Poly (I:C)-induced inflammation requires the activation of toll-like receptor 3/Ca2+/CaMKII/pannexin 1-dependent signaling
Source: Theranostics. 2025 Jan 20;15(6):2470–86. doi: 10.7150/thno.100687 (PMC11840745; doi:10.7150/thno.100687)
Supplement: Supplementary file 1 — Supplementary figures. [file thnov15p2470s1.pdf]

**Poly (I:C)-induced inflammation requires the activation of toll-like receptor 3/$Ca^{2+}$/CaMKII/pannexin 1-dependent signaling**

## SUPPLEMENTARY FIGURES

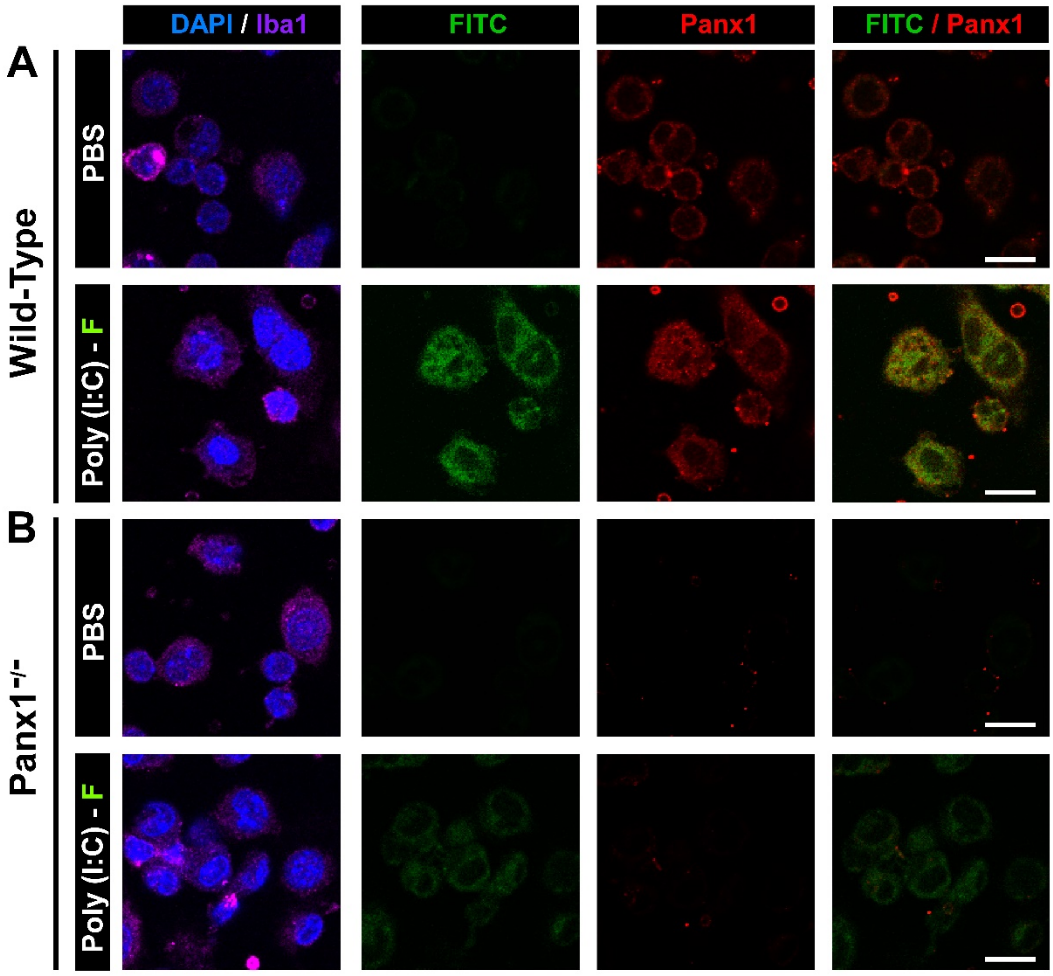


**Figure S1. Poly (I:C) internalization in peritoneal macrophages requires pannexin1 expression**. PBS or Poly (I:C) bound to fluorescein (Poly (I:C) - F) was i.p. injected in wild-type (A) and $Panx1^{-/-}$ (B) mice. Twelve hours later, peritoneal macrophages were extracted, and indirect immunofluorescence was performed to detect Iba-1 and Panx1. Poly (I:C) localization was followed by fluorescein fluorescence (FITC) (n = 3). Bar = 10 µm.

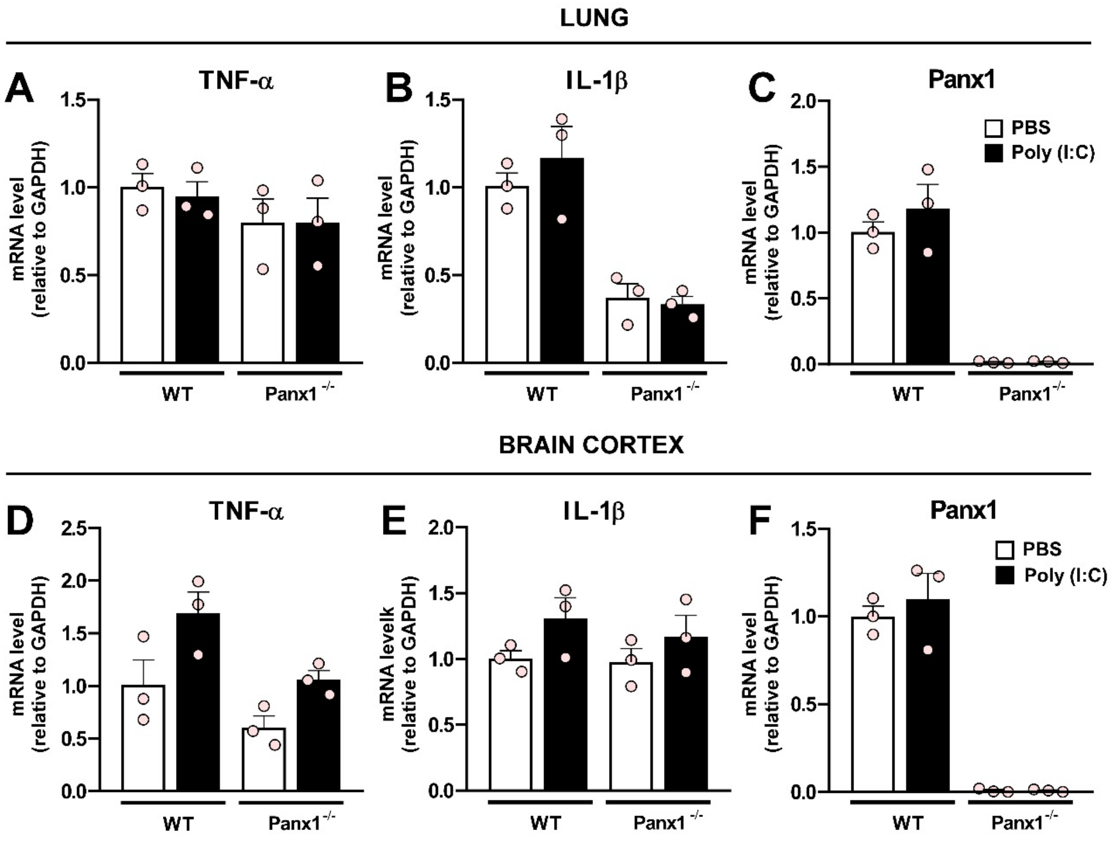


**Figure S2. Intraperitoneal Poly (I:C) injection in mice does not affect the mRNA levels of TNF-α, IL-1β, or Panx1 in the brain cortex or lung**. **A-C**. mRNA levels of TNF-α (A), IL-1β (B), or Panx1 (C) in lung from WT or Panx1$^{-/-}$ mice after 12 h of injection (i.p.) with PBS or 30 µg Poly (I:C) (n = 3). **D-F**. mRNA levels of TNF-α (A), IL-1β (B), or Panx1 (C) in brain cortex from WT or Panx1$^{-/-}$ mice after 12 h of i.p.-injection with PBS or 30 µg Poly (I:C) (n = 3). Each point represents the mean ± SEM. One-way ANOVA followed by Tukey's post hoc test.

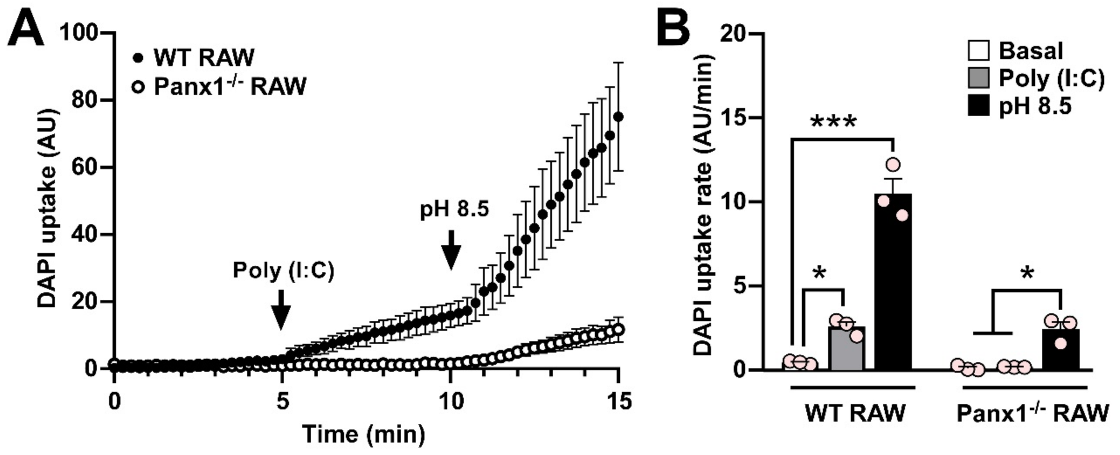


**Figure S3. Poly (I:C) does not increase the membrane permeability to DAPI in pannexin knock-out RAW cells. A.** DAPI uptake in WT RAW and Panx1$^{-/-}$ RAW cells, in response to Poly (I:C) and alkaline pH (8.5). **B**. DAPI uptake rates from A (n = 3). Each point represents the mean ± SEM. one-way ANOVA followed by Tukey's post hoc test. * $p < 0.05$; *** $p < 0.001$**.**

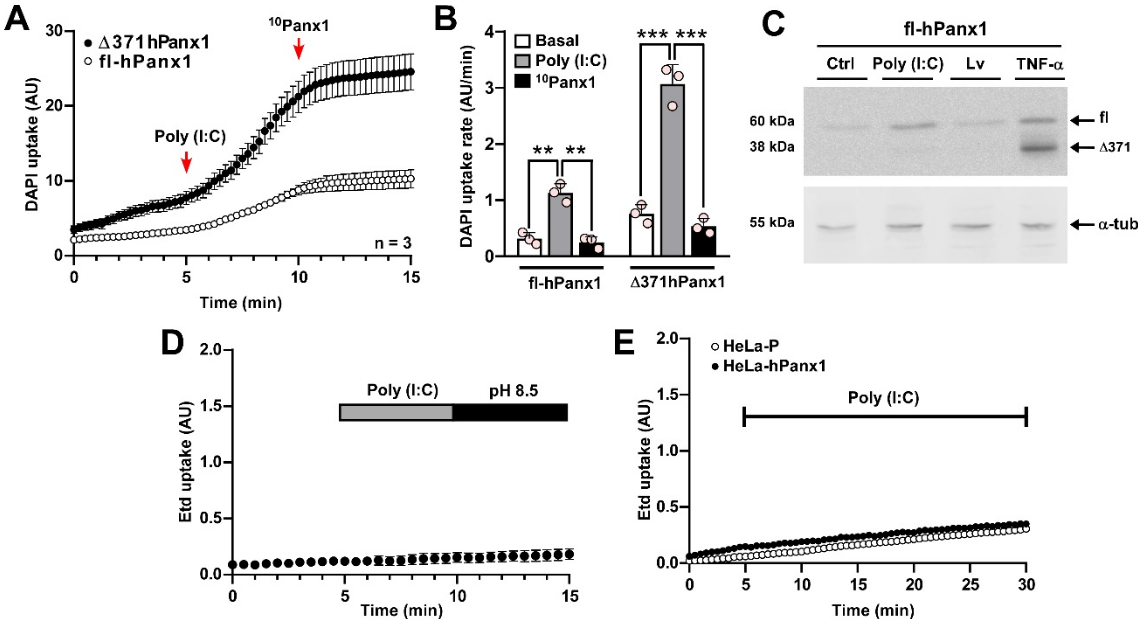


**Figure S4. Poly (I:C) increases the activity of human pannexin1 hemichannels without inducing C-terminal cleavage. A.** Time-lapse DAPI uptake assays in HeLa cells transfected with full-length hPanx1 (fl-hPanx1), or C-terminal cleaved (Δ371hPanx1) in response to 10 µg/mL Poly (I:C) and subsequent application of 200 µM $^{10}$Panx1. **B**. DAPI uptake rates from A. **C**. Western blot analysis of hPanx1 cleavage in response to 6 h application of Poly (I:C), $10^5$ ifu/mL of spike-pseudotyped lentivirus (Lv-spike), or 10 ng/mL TNF-α. α-tubulin was used as the loading control. **D**. $Etd^+$ uptake analysis in HeLa-hPanx1 cells in response to 50 µg/mL Poly (I:C) and subsequent application of alkaline Krebs (pH = 8.5) (n = 3). **E**. $Etd^+$ uptake analysis in HeLa-hPanx1 cells upon long exposure (25 min) to 50 µg/mL Poly (I:C) (n = 3). Each point represents the mean ± SEM. one-way ANOVA followed by Tukey post hoc test. ** $p < 0.01$, *** $p < 0.001$.

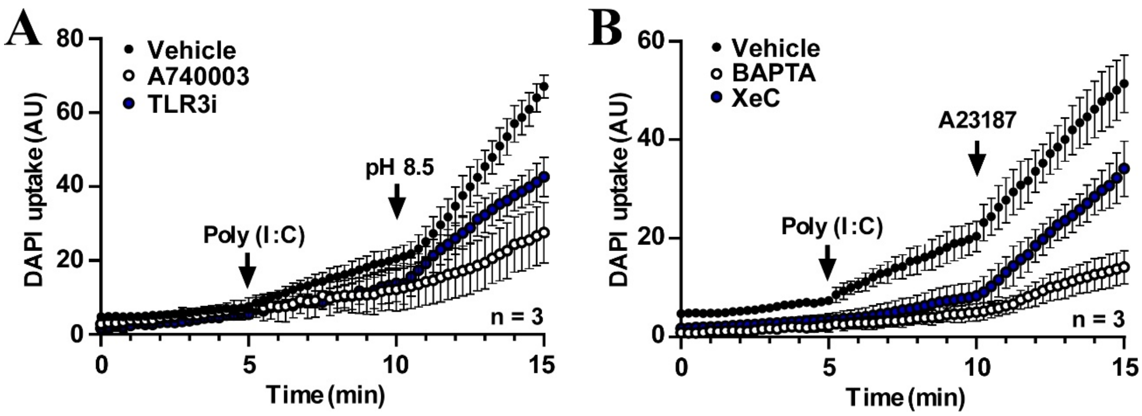


**Figure S5. Poly (I:C)-induced activation of pannexin1 hemichannels depends on toll-like receptor 3, $P2X_7R$, and reticular $Ca^{2+}$ mobilization**. **A**. DAPI uptake curves from RAW cells in response to 10 µg/mL Poly (I:C) (black circles) or co-incubated with 20 µM A740003 (white circles) or 30 µM TLR3i (blue circles) (n = 3). **B**. DAPI uptake curves from RAW cells in response to 10 µg/mL Poly (I:C) or co-incubated with 10 µM BAPTA-AM (white circles) or 5 µM XeC (blue circles). n = 3. Each point represents the mean ± SEM.

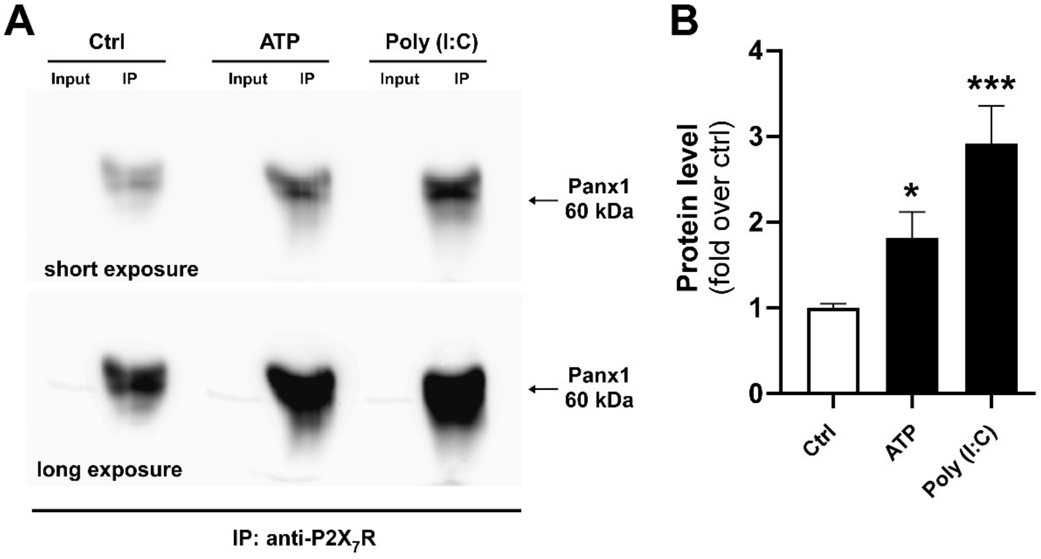


**Figure S6. Evaluation of Panx1-P2X$_7$R interaction by co-immunoprecipitation in RAW cells**. **A**. Western blot analysis showing Panx1 presence in P2X$_7$R-mediated immunoprecipitants (IP) from RAW cells, untreated or incubated with 0.5 mM ATP or 10 µg/mL Poly (I:C). A long exposure (40 min) was used for input visualization under each condition. **B**. Densitometric analysis of Panx1 band intensity from each IP. The quantification of each band was normalized to the control condition ($n = 3$). Bars represent the mean ± SEM. Statistical analysis was performed using one-way ANOVA followed by Tukey's post hoc test. * $p < 0.05$, *** $p < 0.001$.

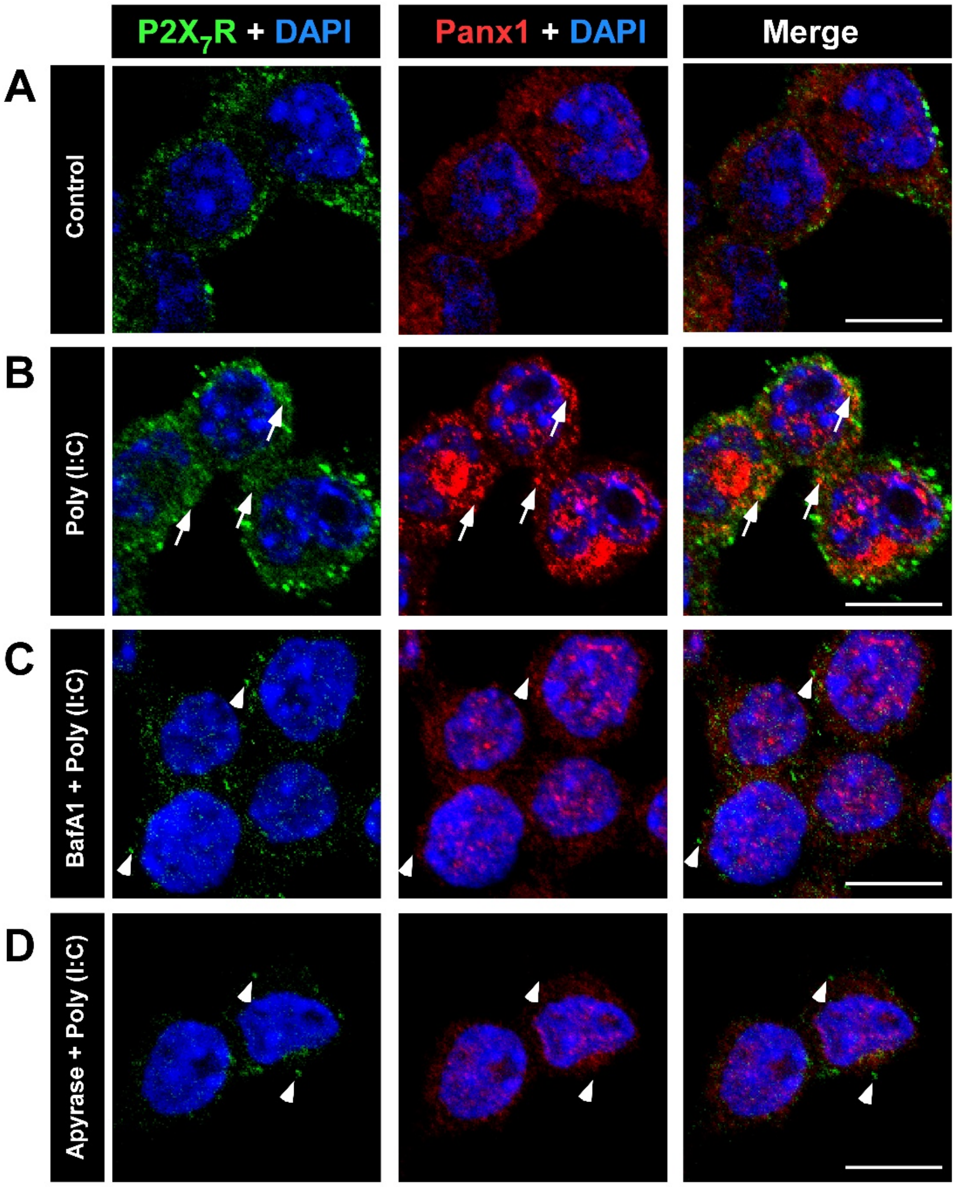


**Figure S7. Poly (I:C) induces pannexin1 and P2X$_7$R endocytosis in RAW cells**. **A-B**. RAW cells were incubated with PBS (A) or 10 µg/mL Poly (I:C) (B) for 30 min and processed for immunofluorescence against Panx1 (red) and P2X$_7$R (green). DAPI was used as a nuclear marker. **C-D**. In separate experiments, RAW cells were preincubated for 15 min with 50 nM bafilomycin A1 (BafA1) (C) or 10 U/mL apyrase (D) before Poly (I:C) application. Arrows indicate regions where Panx1 colocalizes with P2X$_7$R, whereas arrowheads indicate no colocalization. Representative images from at least 3 separate experiments. Bar: 5 µm.

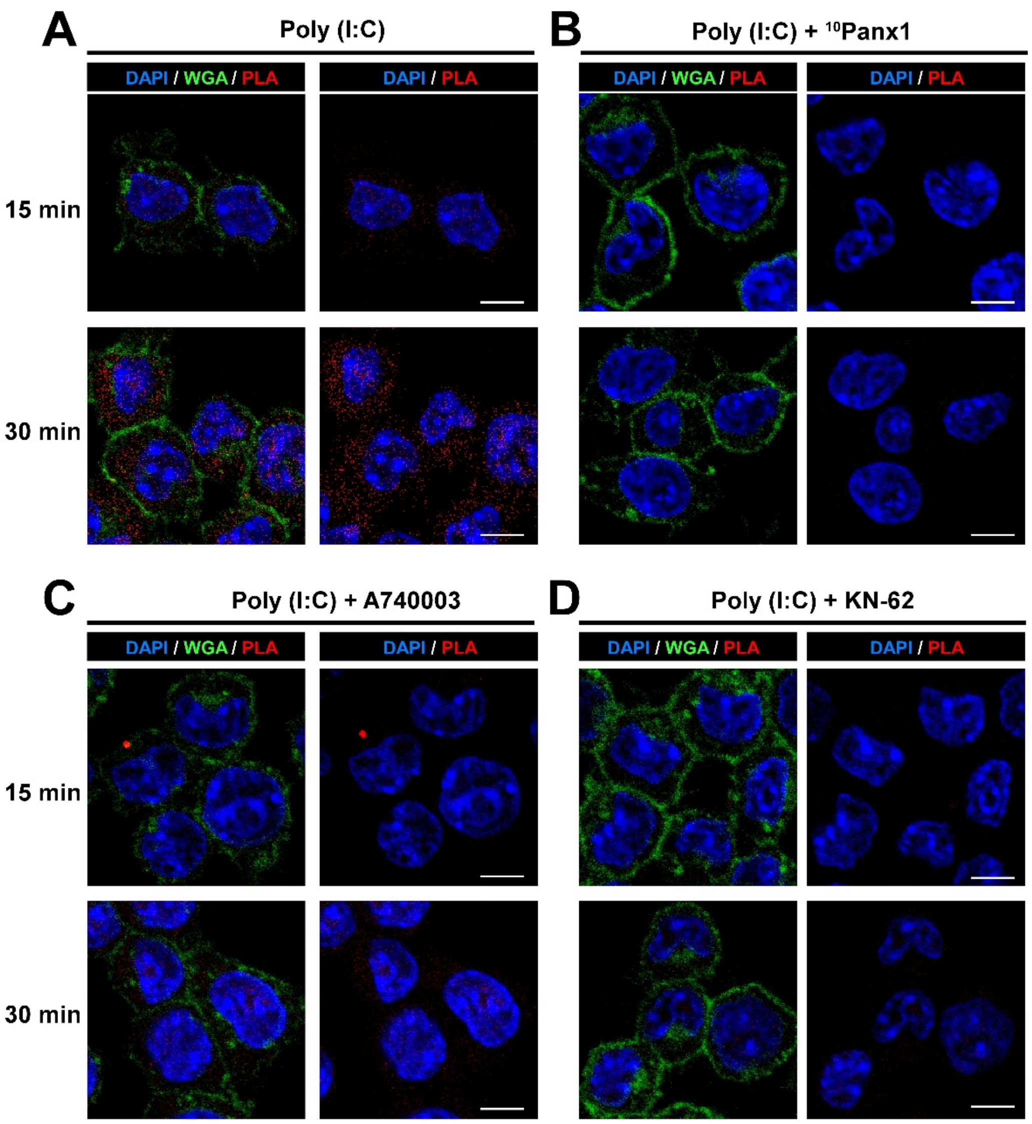


**Figure S8. Panx1-P2X$_7$R interaction in response to Poly (I:C) depends on pannexin1, P2X$_7$R, and CaMKII activities**. Raw cells were 30 min preincubated with PBS (A), 200 µM $^{10}$Panx1 (B), 20 µM A740003 (C), or 10 µM KN-62 (D) and then exposed to 10 µg/mL Poly (I:C) for 15- or 30-min. After fixation, cell membranes were stained with WGA-Alexa fluor 488, and cells were processed to proximity ligation assays (PLA). Representative images from at least 3 separate experiments. Bar: 5 µm.
